# Global trends in esophageal cancer: sex and age disparities in health inequalities from 1990 to 2021, with projections to 2050

**DOI:** 10.3389/fonc.2025.1563570

**Published:** 2025-06-24

**Authors:** Ying Liu, Erman Wu, Fang Cheng, Meng Zhang, Qian Rou, Zenati Nuertai, Maorong Xu, Shanshan Xu, Minghui Li, Lei Zhang, Aheli Nasiroula

**Affiliations:** ^1^ Special Needs Comprehensive Department, Affiliated Tumor Hospital of Xinjiang Medical University, Urumqi, Xinjiang, China; ^2^ State Key Laboratory of Pathogenesis, Prevention and Treatment of High Incidence Diseases in Central Asia, Xinjiang Medical University, Urumqi, China; ^3^ Department of Neurosurgery, The First Affiliated Hospital of Xinjiang Medical University, Urumqi, Xinjiang, China; ^4^ Department of Computer Science and Information Technologies, Elviña, University of A Coruña, A Coruña, Spain

**Keywords:** esophageal cancer, global burden of disease, sex disparities, age disparities, sociodemographic index, health inequalities, disease projections

## Abstract

**Background:**

Esophageal cancer remains one of the deadliest cancers globally, highlighting significant health challenges and socioeconomic disparities. This study aims to measure its global burden, assess disparities by sex, age, and region, and evaluate health inequalities, with projections to 2050. The goal is to provide evidence to guide resource allocation and reduce the disease burden.

**Methods:**

Using data from the Global Burden of Disease (GBD) 2021 study, we analyzed trends in prevalence, incidence, mortality, and Disability-Adjusted Life Years (DALYs) across sexes, age groups, and 204 countries and territories. Age-standardized rates (ASR) were calculated to account for population age structures. Trends over time were assessed using the estimated annual percentage change (EAPC). Health inequalities were evaluated using the Slope Index of Inequality (SII) and Concentration Index (CI). Future burdens were projected using Bayesian Age-Period-Cohort (BAPC) models.

**Results:**

From 1990 to 2021, esophageal cancer cases increased: prevalence from 551.62 to 1004.2 thousand, incidence from 354.73 to 576.53 thousand, mortality from 356.26 to 538.6 thousand, and DALYs from 9753.57 to 12999.26 thousand. However, age-standardized rates declined: prevalence from 13.34 to 11.47, incidence from 8.86 to 6.65, mortality from 9.02 to 6.25, and DALYs from 235.32 to 148.56 per 100,000 people. The burden rises sharply after age 40, with males and low-SDI regions experiencing higher burdens. Health inequalities widened, with the SII for prevalence increasing from 2.52 to 5.67, and for deaths from 1.45 to 2.94. West Africa, North Europe, and North America saw rising prevalence rates, while East Asia showed a declining trend. A decreasing trend is observed in most countries and regions worldwide, particularly in East Asia, with projections suggesting a continued decline in the future.

**Conclusion:**

Although projections indicate a decreasing trend, health inequalities have intensified. Regions such as West Africa, North Europe, and North America are experiencing rising prevalence. To address these disparities, targeted interventions, enhanced healthcare access, and preventive measures in high-burden areas are essential to reduce the global burden and advance health equity.

## 1 Introduction

Esophageal cancer remains a significant challenge to global public health which presents not just medical difficulties but also significant societal and economic concerns (1, 2). It not only deteriorates patient’s quality of life but also places a significant strain on healthcare systems and economic progress (3, 4). As demographics shift and lifestyle evolve, the prevalence, incidence and mortality patterns of esophageal cancer exhibit complex and variable trends, necessitating a more nuanced comprehension of the disease (5–7).

The etiology of esophageal cancer is multifactorial, encompassing genetic, environmental, and dietary factors (8–10), which vary globally and contribute to the uneven distribution of the disease across geographies, age groups, genders, and socioeconomic status (11, 12). East Asia, especially China, exhibits the highest incidence and mortality rates of esophageal cancer, likely attributable to dietary factors (13, 14). High consumption of extremely hot beverages and foods may harm the esophageal lining, escalating cancer risk (15). Moreover, a diet deficient in fruits and vegetables and rich in salted, pickled, and smoked foods, prevalent in certain Chinese regions, could also contribute to the heightened risk (16, 17).

Age is a critical determinant, with esophageal cancer rates reaching their zenith among the elderly, frequently associated with coexisting chronic diseases (18–20). The incidence is higher in males compared to females (21), potentially because of their increased engagement in risky behaviors such as smoking, excessive alcohol intake, and exposure to occupational hazards (22).

Disparities in healthcare resources significantly affect the burden of esophageal cancer. In low- and middle-income areas, the dearth of medical resources and constrained diagnostic and therapeutic capabilities lead to unfavorable patient outcomes and a substantial financial strain on healthcare systems (23, 24).

Considering these issues, thorough research into the disease burden of esophageal cancer, including its geographical distribution, age and gender disparities, and the allocation of healthcare resources, is essential (6, 7, 18). This study endeavors to scrutinize global disease burden data from 1990 to 2021, evaluating the progression and regional challenges of esophageal cancer. It also endeavors to forecast the disease’s trajectory from 2022 to 2050. The goal is to provide scientific evidence and formulate policy recommendations for global esophageal cancer prevention and management, thereby lessening its broader impact on individuals and society.

## 2 Methods

### 2.1 Data sources

The Global Burden of Disease (GBD) 2021 study, conducted by the Institute for Health Metrics and Evaluation (IHME) at the University of Washington, provides an exhaustive analysis of the burden imposed by a wide array of diseases and injuries on the global health landscape (25). This comprehensive assessment is grounded in the examination of critical health metrics, including prevalence, which denotes the total volume of existing cases at a given time; incidence, signifying the frequency of new cases over a specified period; and mortality, which indicates the number of fatalities attributed to these conditions. To provide a more holistic view of the impact on population health, the GBD study also quantifies Disability-Adjusted Life Years (DALYs), a metric that amalgamates the years of life lost due to early death with the years lived with disability. To facilitate meaningful comparisons across populations with varying age structures, age-standardized rates are calculated. Additionally, the Socio-Demographic Index (SDI) is employed as a multifaceted measure of developmental status, offering a framework for understanding health outcomes within diverse societal contexts. Access to the data can be obtained through the IHME’s official website at https://www.healthdata.org/.

The GBD collection team has established comprehensive data privacy measures to ensure ethical compliance (https://www.healthdata.org/data-tools-practices/data-practices). Data transparency is maintained by adhering to the GATHER guidelines and providing detailed data source descriptions on the GHDx platform. Individual privacy is safeguarded through strict confidentiality measures and compliance with data agreements with providers. Data management practices include secure storage on controlled-access servers, role-based access control, and adherence to data use agreements. Staff receive regular training on handling human subjects’ data, and all activities comply with relevant laws and regulations, including GDPR requirements for health data. Data protection impact assessments (DPIAs) are conducted for high-risk data processing activities, and robust technical and administrative measures are implemented to ensure data integrity and security. These practices collectively guarantee that GBD data is managed ethically and securely throughout its lifecycle.

In the 9th edition of the International Classification of Diseases (ICD-9), esophageal cancer is categorized under codes 150-150.9, while in the 10th revision (ICD-10), it is classified under codes C15-C15.9, D00.1(**Supplementary Table S6**). ICD-9 to ICD-10 coding differences for esophageal cancer were harmonized using the WHO General Equivalence Mappings (GEMs) to ensure consistency across study periods.

### 2.2 Statistical analysis

We employed age-standardized rates (ASR) of prevalence, incidence, mortality and DALYs to evaluate variations by sex, age, year, and region. The analysis spanned 21 geographical regions and covered a range of SDI levels across 204 countries and territories. To measure the trend over time, the estimated annual percentage change (EAPC) in ASR from 1990 to 2021, assuming a linear association between the natural logarithm of ASR and the calendar year, as shown by the equation.


Y = α + βX + ϵ

Where Y is the natural logarithm of ASR, X denotes the year, α is the intercept, β represents the yearly change in the natural logarithm of ASR, and ϵis the error term. The EAPC is computed as.


$$EAPC\;=\;100\;\times \;\left(e^{\beta }-\;1\right)$$

We extracted 95% confidence intervals from the linear regression model, with a positive EAPC suggesting an upward trend and a negative EAPC indicating a downward trend.

### 2.3 Cross-country inequality analysis

Adhering to the World Health Organization’s (WHO) guidelines, two key measures are used to evaluate income-related health inequalities: the Slope Index of Inequality (SII) and the Concentration Index (CI) (26). Health inequalities are defined as systematic, avoidable, and unfair disparities in health outcomes observed between different population groups, within social groups of the same population, or as a gradient across a population ranked by social position (27). Absolute health inequality quantifies the magnitude of the difference in health between subgroups, measured by the actual difference in health outcomes, using the same unit as the health indicator (https://www.who.int/data/inequality-monitor/tools-resources/book_2024).

To calculate the SII, the population is ranked by the socio-economic status, with the most disadvantaged at rank 0 and the most advantage at rank 1. The Health Equity Assessment Toolkit (HEAT) plus software is used to regress the health indicator of interest against the midpoint of this ranked distribution. The SII is then computed as the difference between the predicted indicator values at the two extremes of the ranking.


SII = |v1 – v0|

Where v1 is the value at rank 1 (most advantaged) and v0 is the value at rank 0 (most disadvantaged). The sign of the difference depends on whether the indicator is favorable or unfavorable; a larger value at the higher rank indicates a favorable outcome, while a larger value at the lower rank indicates an unfavorable outcome.

The Concentration Index (CI) is a measure used to assess health inequality across socio-economic subgroups within a population. It is calculated by constructing a Lorenz curve that plots the cumulative percentage of the health indicator against the cumulative percentage of the population, stratified by socio-economic status, and then determining the area between this curve and the line of perfect equality.

The CI is normalized to range from -1 to +1, with 0 indicating equal distribution, positive values indicating concentration among the advantaged, and negative values indicating concentration among the disadvantaged. To obtain the Relative Concentration Index (RCI), the Absolute Concentration Index (ACI) is divided by the mean health indicator value (m) and multiplied by 100.


RCI = (ACI/m) * 100

It provides a percentage measure of inequality relative to the global average. This approach allows for the quantification and comparison of health disparities, with higher absolute RCI values indicating greater degrees of inequality.

### 2.4 Projection analysis

The projection analysis, utilizing Bayesian Age-Period-Cohort (BAPC) models, is a sophisticated approach designed to forecast future trends in health-related data (28). Our Bayesian Age-Period-Cohort (BAPC) model, built with the Integrated Nested Laplace Approximation (INLA) package, disentangles temporal trends into age, period, and cohort effects. This is achieved through 5th-order B-splines (gf=5) and intrinsic Gaussian Markov Random Field priors. The model posits a Poisson likelihood and leverages INLA for effective posterior estimation. For validation, we conduct retrospective comparisons (1990 - 2021), Geweke diagnostics for convergence, and sensitivity analyses with alternative priors. Our predictive projections (2022 - 2050) are age-standardized via WHO population weights. The BAPC models are not only capable of generating age-specific incidence and mortality rates but also of producing age-standardized projected rates. This is particularly valuable for adjusting for differences in population age structures, thereby allowing for more accurate comparisons over time and across different populations.

## 3 Result

### 3.1 Global disease burden of esophageal cancer from 1990 to 2021 and projection from 2022 to 2050

**Figure 1:**
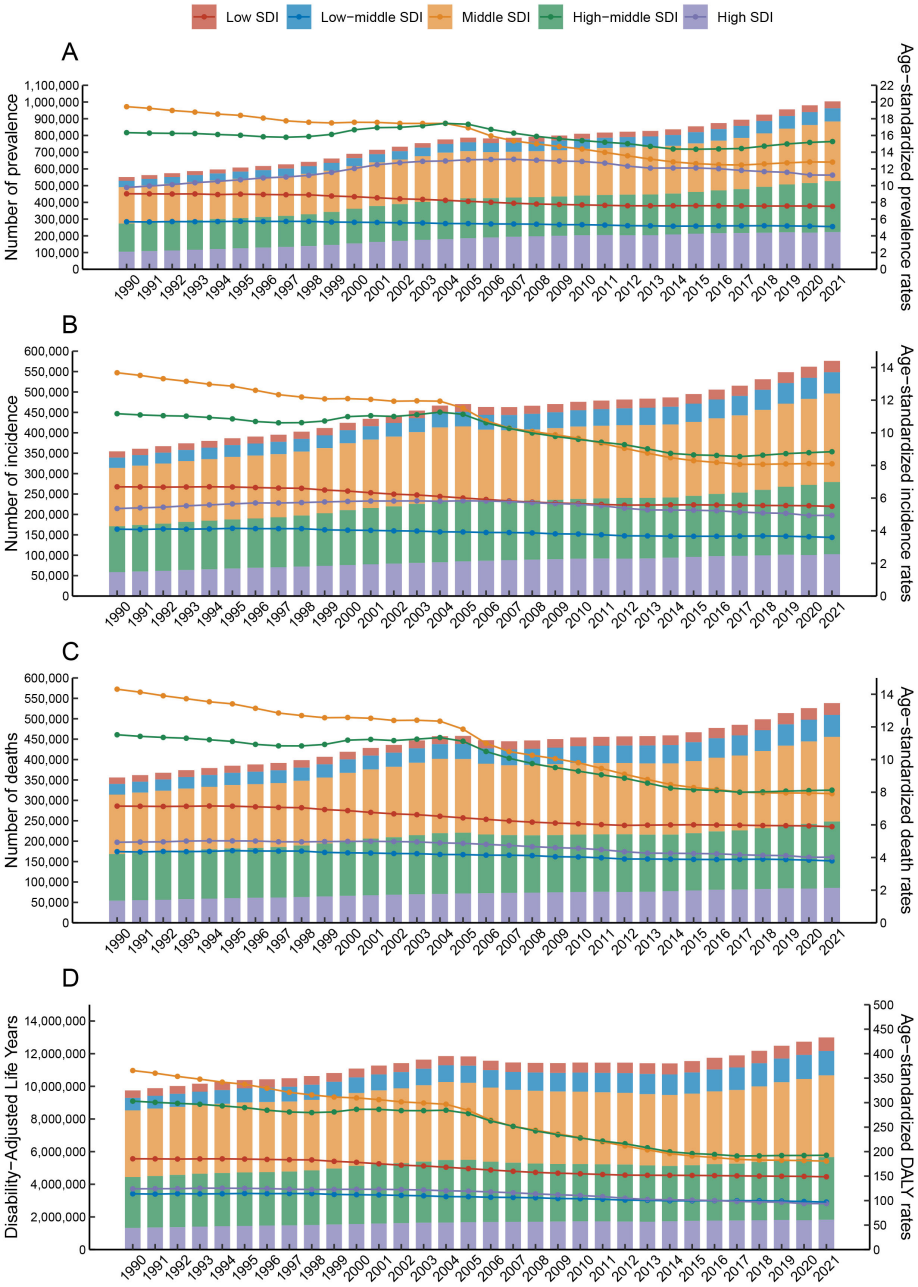
Disease burden of esophageal cancer across five SDI quintiles from 1990 to 2021, analyzed by number and age-standardized rates. The X-axis represents the years, while the left Y-axis indicates the number of (**(A)** prevalence, **(B)** incidence, **(C)** deaths, **(D)** Disability-Adjusted Life Years). The right Y-axis shows the age-standardized rate for (**(A)** prevalence, **(B)** incidence, **(C)** deaths, **(D)** Disability-Adjusted Life Years).

From 1990 to 2021, esophageal cancer saw a rise in the number of prevalence, incidence, mortality, and DALYs, with increases from 551.62 (95% UI, 493.22-605) thousand to 1004.2 (95% UI, 888.17-1120.96) thousand, 354.73 (95% UI, 317.51-388.91) thousand to 576.53 (95% UI, 509.49-645.65) thousand, 356.26 (95% UI, 319.36-390.15) thousand to 538.6 (95% UI, 475.94-603.41) thousand, and 9753.57 (95% UI, 8719.32-10739.56) thousand to 12999.26 (95% UI, 11522.86-14605.27) thousand, respectively (**Figure 1**, **Tables 1**, **2**, **3**, **Supplementary Table S1**). Conversely, age-standardized rates, adjusted for population age structures, showed a slight decrease: prevalence from 13.34 (95% UI, 11.94-14.61) to 11.47 (95% UI, 10.15-12.80), incidence from 8.86 (95% UI, 7.96-9.69) to 6.65 (95% UI, 5.88-7.45), mortality from 9.02 (95% UI, 8.11-9.87) to 6.25 (95% UI, 5.53-7.00), and DALYs from 235.32 (95% UI, 210.52-258.68) to 148.56 (95% UI, 131.71-166.82) per 100,000 people (**Figure 1**; **Tables 1**-**3**, **Supplementary Table S1**). This trend indicates that despite the growing burden of esophageal cancer, there has been a modest improvement in the equalization of rates when demographic shifts are considered.

**Table 1:** Esophageal cancer prevalence in 1990 and 2021, and the associated changes from 1990 to 2021, by geographic region.

Characteristics	1990		2021		1990-2021
	Prevalence no.×10^3^ (95% UI)	Age-standardized prevalence rates (95% UI)	Prevalence no.×10^3^ (95% UI)	Age-standardized prevalence rates (95% UI)	EAPC of age-standardized prevalence rates (95% CI)
Global	551.62 (493.22-605)	13.34 (11.94-14.61)	1004.2 (888.17-1120.96)	11.47 (10.15-12.8)	-0.64 (-0.79–0.5)
Low SDI	22.37 (18.46-25.2)	9.03 (7.51-10.17)	41.84 (35.42-48.31)	7.53 (6.42-8.66)	-0.74 (-0.81–0.67)
Low-middle SDI	37.99 (34.49-43.74)	5.68 (5.14-6.52)	78.27 (70.78-90.06)	5.1 (4.62-5.86)	-0.42 (-0.46–0.38)
Middle SDI	217.38 (181.67-251.98)	19.44 (16.25-22.47)	356.8 (297.6-425.22)	12.82 (10.72-15.25)	-1.63 (-1.77–1.48)
High-middle SDI	168.4 (147.15-189.12)	16.34 (14.29-18.34)	304.47 (249.66-368.83)	15.27 (12.52-18.51)	-0.41 (-0.57–0.24)
High SDI	105.26 (101.73-107.56)	9.78 (9.47-9.99)	222.45 (208.69-231.82)	11.26 (10.62-11.71)	0.48 (0.19-0.78)
Oceania	0.09 (0.07-0.12)	2.8 (2.14-3.71)	0.2 (0.16-0.26)	2.45 (1.94-3.08)	-0.44 (-0.47–0.41)
Australasia	1.73 (1.64-1.83)	7.37 (6.99-7.75)	3.95 (3.58-4.23)	7.62 (6.95-8.14)	0.16 (-0.01-0.32)
East Asia	318.75 (264.55-371.43)	34.11 (28.32-39.61)	559.32 (449.55-685.97)	24.74 (19.92-30.25)	-1.31 (-1.51–1.12)
South Asia	35.43 (31.52-42.95)	5.49 (4.88-6.64)	75.49 (66.65-89.74)	4.8 (4.24-5.72)	-0.67 (-0.77–0.57)
Southeast Asia	10.67 (8.93-12.54)	3.81 (3.21-4.46)	26.44 (22.69-30.6)	3.74 (3.22-4.31)	-0.1 (-0.14–0.06)
Central Asia	8.54 (8.17-8.91)	17.58 (16.79-18.35)	5.22 (4.63-5.83)	6.02 (5.37-6.69)	-3.52 (-3.71–3.33)
Eastern Europe	18.72 (18.33-19.12)	6.54 (6.4-6.68)	17.45 (15.74-18.95)	5.16 (4.65-5.61)	-0.93 (-1.2–0.66)
Central Europe	6.34 (6.09-6.62)	4.21 (4.04-4.39)	8.63 (7.9-9.32)	4.26 (3.9-4.61)	-0.18 (-0.32–0.03)
Western Europe	43.79 (42.52-44.81)	8.13 (7.9-8.32)	76.36 (71.33-79.5)	9.24 (8.72-9.58)	0.56 (0.37-0.74)
High-income Asia Pacific	32.28 (30.85-33.5)	15.59 (14.87-16.17)	76.56 (69.63-80.76)	17.79 (16.48-18.73)	0.44 (0.07-0.81)
High-income North America	23.89 (22.97-24.38)	7.17 (6.93-7.31)	49.55 (47.13-51.31)	7.96 (7.6-8.23)	0.29 (0.08-0.5)
Andean Latin America	0.53 (0.46-0.6)	2.52 (2.22-2.87)	1.08 (0.88-1.35)	1.82 (1.48-2.26)	-1.12 (-1.25–0.99)
Tropical Latin America	9.1 (8.8-9.35)	9.3 (8.94-9.56)	19.33 (18.38-20.08)	7.33 (6.96-7.62)	-0.77 (-0.86–0.69)
Southern Latin America	4.44 (4.25-4.62)	9.53 (9.12-9.93)	4.71 (4.4-5.04)	5.45 (5.11-5.83)	-1.8 (-2.01–1.59)
Central Latin America	2.86 (2.79-2.92)	3.34 (3.23-3.41)	5.43 (4.85-6.12)	2.14 (1.91-2.41)	-1.54 (-1.62–1.46)
Caribbean	1.41 (1.33-1.5)	5.38 (5.06-5.72)	2.96 (2.6-3.35)	5.47 (4.79-6.19)	0.36 (0.22-0.49)
North Africa and Middle East	6.68 (5.26-7.68)	3.69 (2.94-4.23)	15.91 (13.49-17.86)	3.36 (2.88-3.78)	-0.4 (-0.49–0.31)
Central Sub-Saharan Africa	3.54 (2.58-4.51)	14.11 (10.4-17.72)	6.88 (5.01-8.95)	11.13 (8.15-14.43)	-0.9 (-1–0.79)
Eastern Sub-Saharan Africa	14.77 (11.99-17)	18.11 (14.85-20.85)	27.46 (22.78-33.15)	14.8 (12.35-17.81)	-0.82 (-0.88–0.76)
Southern Sub-Saharan Africa	4.78 (4.3-5.49)	16.16 (14.44-18.62)	9.4 (8.58-10.32)	15.08 (13.76-16.5)	-0.6 (-1.01–0.18)
Western Sub-Saharan Africa	3.27 (2.62-4.02)	3.52 (2.84-4.3)	11.87 (8.84-14.3)	5.59 (4.18-6.7)	2.04 (1.84-2.25)

**Table 2:** Esophageal cancer incidence in 1990 and 2021, and the associated changes from 1990 to 2021, by geographic region.

Characteristics	1990		2021		1990-2021
	Incidence no.×10^3^ (95% UI)	Age-standardized incidence rates (95% UI)	Incidence no.×10^3^ (95% UI)	Age-standardized incidence rates (95% UI)	EAPC of age-standardized incidence rates (95% CI)
Global	354.73 (317.51-388.91)	8.86 (7.96-9.69)	576.53 (509.49-645.65)	6.65 (5.88-7.45)	-1.12 (-1.25–1)
Low SDI	15.3 (12.75-17.27)	6.69 (5.58-7.51)	27.96 (23.83-32.18)	5.49 (4.7-6.32)	-0.8 (-0.87–0.72)
Low-middle SDI	25.33 (22.89-29.04)	4.1 (3.68-4.7)	52.1 (47.17-59.93)	3.59 (3.24-4.15)	-0.5 (-0.55–0.46)
Middle SDI	143.09 (120.41-165.38)	13.68 (11.49-15.77)	216.95 (182.21-258.45)	8.1 (6.78-9.62)	-1.95 (-2.1–1.81)
High-middle SDI	112.27 (98.61-125.91)	11.17 (9.85-12.49)	176.77 (145.14-214.12)	8.84 (7.26-10.7)	-0.97 (-1.14–0.8)
High SDI	58.6 (56.15-60.08)	5.36 (5.14-5.49)	102.51 (95.22-107.35)	4.94 (4.63-5.16)	-0.34 (-0.5–0.19)
Oceania	0.06 (0.05-0.08)	2.11 (1.65-2.75)	0.13 (0.1-0.17)	1.81 (1.43-2.28)	-0.51 (-0.53–0.48)
Australasia	1.05 (1-1.11)	4.47 (4.23-4.72)	2.2 (1.98-2.36)	4.05 (3.68-4.33)	-0.4 (-0.49–0.31)
East Asia	210.69 (175.69-244.7)	24.23 (20.24-28.01)	327.71 (263.65-401.88)	14.83 (11.94-18.09)	-1.85 (-2.06–1.64)
South Asia	23.22 (20.62-28.08)	3.93 (3.45-4.75)	50.08 (44.23-59.87)	3.36 (2.95-4.03)	-0.75 (-0.85–0.64)
Southeast Asia	6.96 (5.84-8.15)	2.69 (2.26-3.14)	16.16 (13.98-18.58)	2.42 (2.11-2.76)	-0.4 (-0.44–0.36)
Central Asia	5.95 (5.67-6.22)	12.77 (12.15-13.35)	3.57 (3.19-3.97)	4.42 (3.98-4.9)	-3.49 (-3.67–3.32)
Eastern Europe	12.4 (12.11-12.64)	4.36 (4.25-4.44)	10.71 (9.69-11.62)	3.09 (2.79-3.35)	-1.33 (-1.54–1.11)
Central Europe	4.32 (4.16-4.5)	2.89 (2.77-3)	5.76 (5.28-6.21)	2.73 (2.5-2.95)	-0.43 (-0.55–0.3)
Western Europe	27.62 (26.59-28.33)	4.92 (4.75-5.04)	38.42 (35.45-40.22)	4.26 (4-4.44)	-0.48 (-0.57–0.39)
High-income Asia Pacific	13.28 (12.68-13.83)	6.53 (6.23-6.81)	25.55 (22.82-27.08)	5.49 (5-5.8)	-0.64 (-0.84–0.45)
High-income North America	14.12 (13.44-14.47)	4.12 (3.93-4.21)	27.33 (25.62-28.41)	4.2 (3.96-4.36)	-0.03 (-0.19-0.13)
Andean Latin America	0.39 (0.34-0.44)	1.96 (1.73-2.23)	0.8 (0.66-0.99)	1.38 (1.14-1.7)	-1.2 (-1.31–1.08)
Tropical Latin America	6.09 (5.84-6.26)	6.63 (6.3-6.83)	12.77 (12.08-13.28)	4.91 (4.64-5.11)	-0.94 (-1.02–0.86)
Southern Latin America	3.28 (3.13-3.42)	7.17 (6.85-7.49)	3.43 (3.19-3.67)	3.89 (3.62-4.15)	-1.98 (-2.19–1.77)
Central Latin America	2.03 (1.96-2.08)	2.57 (2.46-2.63)	3.81 (3.4-4.29)	1.54 (1.37-1.73)	-1.78 (-1.86–1.7)
Caribbean	1 (0.94-1.06)	3.91 (3.69-4.14)	1.95 (1.71-2.21)	3.6 (3.17-4.08)	0 (-0.13-0.14)
North Africa and Middle East	4.31 (3.44-4.94)	2.59 (2.1-2.97)	8.68 (7.37-9.77)	1.99 (1.71-2.22)	-0.93 (-0.98–0.88)
Central Sub-Saharan Africa	2.4 (1.78-3.02)	10.59 (7.92-13.19)	4.54 (3.32-5.88)	8.26 (6.03-10.61)	-0.94 (-1.04–0.84)
Eastern Sub-Saharan Africa	10.17 (8.34-11.73)	13.56 (11.19-15.62)	18.38 (15.33-22.11)	10.93 (9.14-13.09)	-0.88 (-0.95–0.81)
Southern Sub-Saharan Africa	3.1 (2.77-3.57)	11.23 (9.98-13.05)	6.41 (5.85-7.01)	11.01 (10.06-11.99)	-0.43 (-0.9-0.04)
Western Sub-Saharan Africa	2.31 (1.87-2.81)	2.65 (2.16-3.21)	8.14 (6.1-9.76)	4.22 (3.15-5.02)	2.07 (1.86-2.28)

**Table 3:** Esophageal cancer death in 1990 and 2021, and the associated changes from 1990 to 2021, by geographic region.

Characteristics	1990		2021		1990-2021
	Death no.×10^3^ (95% UI)	Age-standardized death rates (95% UI)	Death no.×10^3^ (95% UI)	Age-standardized death rates (95% UI)	EAPC of age-standardized death rates (95% CI)
Global	356.26 (319.36-390.15)	9.02 (8.11-9.87)	538.6 (475.94-603.41)	6.25 (5.53-7)	-1.41 (-1.55–1.27)
Low SDI	15.83 (13.2-17.87)	7.15 (5.97-8.01)	28.92 (24.61-33.45)	5.89 (5.02-6.8)	-0.78 (-0.85–0.7)
Low-middle SDI	26.14 (23.62-30.03)	4.36 (3.92-5.02)	53.72 (48.51-61.81)	3.79 (3.42-4.39)	-0.53 (-0.57–0.48)
Middle SDI	145.57 (123.68-168.24)	14.31 (12.19-16.45)	207.63 (174.86-246.5)	7.91 (6.65-9.34)	-2.18 (-2.34–2.02)
High-middle SDI	114.33 (100.75-128.04)	11.52 (10.19-12.87)	162.43 (134.26-195.47)	8.13 (6.72-9.77)	-1.38 (-1.58–1.18)
High SDI	54.24 (51.96-55.65)	4.93 (4.73-5.06)	85.65 (79.16-89.95)	4.02 (3.75-4.2)	-0.78 (-0.89–0.67)
Oceania	0.06 (0.05-0.08)	2.29 (1.8-2.95)	0.13 (0.11-0.17)	1.95 (1.54-2.45)	-0.53 (-0.55–0.5)
Australasia	1.02 (0.97-1.08)	4.34 (4.11-4.6)	2.05 (1.85-2.2)	3.68 (3.33-3.94)	-0.64 (-0.72–0.57)
East Asia	213.97 (179.12-247.92)	25.43 (21.28-29.31)	302.58 (243.36-368.74)	13.91 (11.23-16.84)	-2.23 (-2.46–1.99)
South Asia	23.83 (21.09-28.89)	4.17 (3.66-5.06)	51.54 (45.65-61.69)	3.54 (3.12-4.26)	-0.77 (-0.87–0.66)
Southeast Asia	7.11 (5.96-8.31)	2.83 (2.39-3.3)	15.83 (13.72-18.15)	2.44 (2.13-2.78)	-0.55 (-0.6–0.51)
Central Asia	6.28 (5.98-6.57)	13.68 (13-14.33)	3.74 (3.34-4.16)	4.74 (4.28-5.25)	-3.49 (-3.66–3.32)
Eastern Europe	12.47 (12.18-12.7)	4.41 (4.3-4.49)	10.31 (9.38-11.16)	2.94 (2.68-3.19)	-1.53 (-1.72–1.33)
Central Europe	4.48 (4.31-4.66)	3.02 (2.9-3.13)	5.93 (5.44-6.39)	2.76 (2.54-2.98)	-0.53 (-0.65–0.41)
Western Europe	27.29 (26.18-28.01)	4.8 (4.62-4.92)	34.4 (31.52-36.12)	3.65 (3.41-3.81)	-0.95 (-1.02–0.87)
High-income Asia Pacific	10.3 (9.8-10.77)	5.13 (4.87-5.37)	16.91 (15.03-17.97)	3.42 (3.1-3.62)	-1.43 (-1.52–1.34)
High-income North America	12.93 (12.27-13.26)	3.73 (3.55-3.82)	23.96 (22.39-24.95)	3.62 (3.4-3.76)	-0.2 (-0.33–0.06)

**Figure 2:**
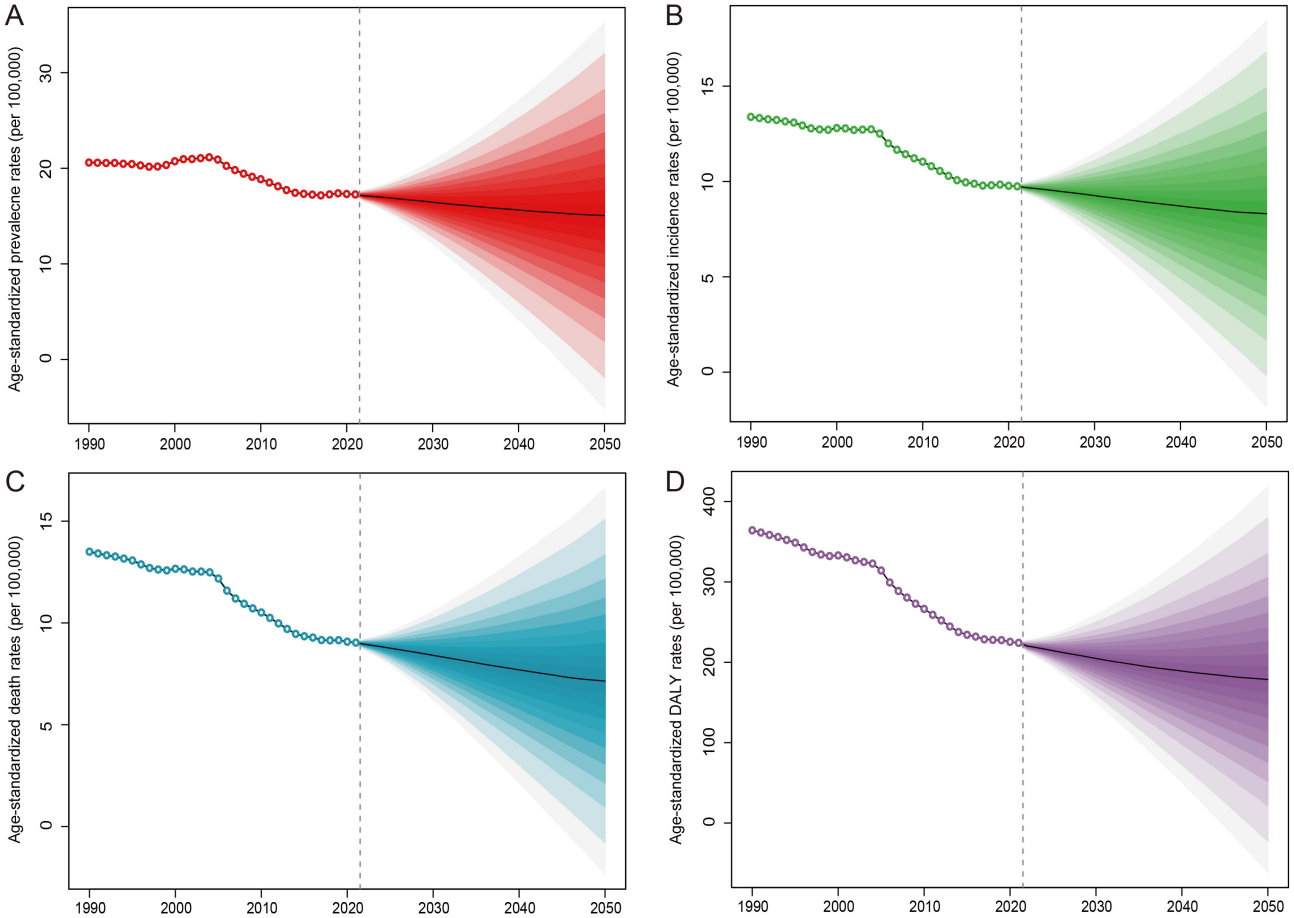
Forecasting the disease burden of esophageal cancer from 2022 to 2050. The X-axis represents the years, while the left Y-axis indicates the age-standardized rate for (**(A)** prevalence, **(B)** incidence, **(C)** deaths, **(D)** Disability-Adjusted Life Years).

Forecasts for the period from 2022 to 2050 suggest a continued, albeit modest, decline in age-standardized rates of esophageal cancer prevalence, incidence, mortality, and DALYs globally (**Figure 2**).

### 3.2 Global disease burden of esophageal cancer from 1990 to 2021: analysis by gender disparity and GBD region

**Figure 3:**
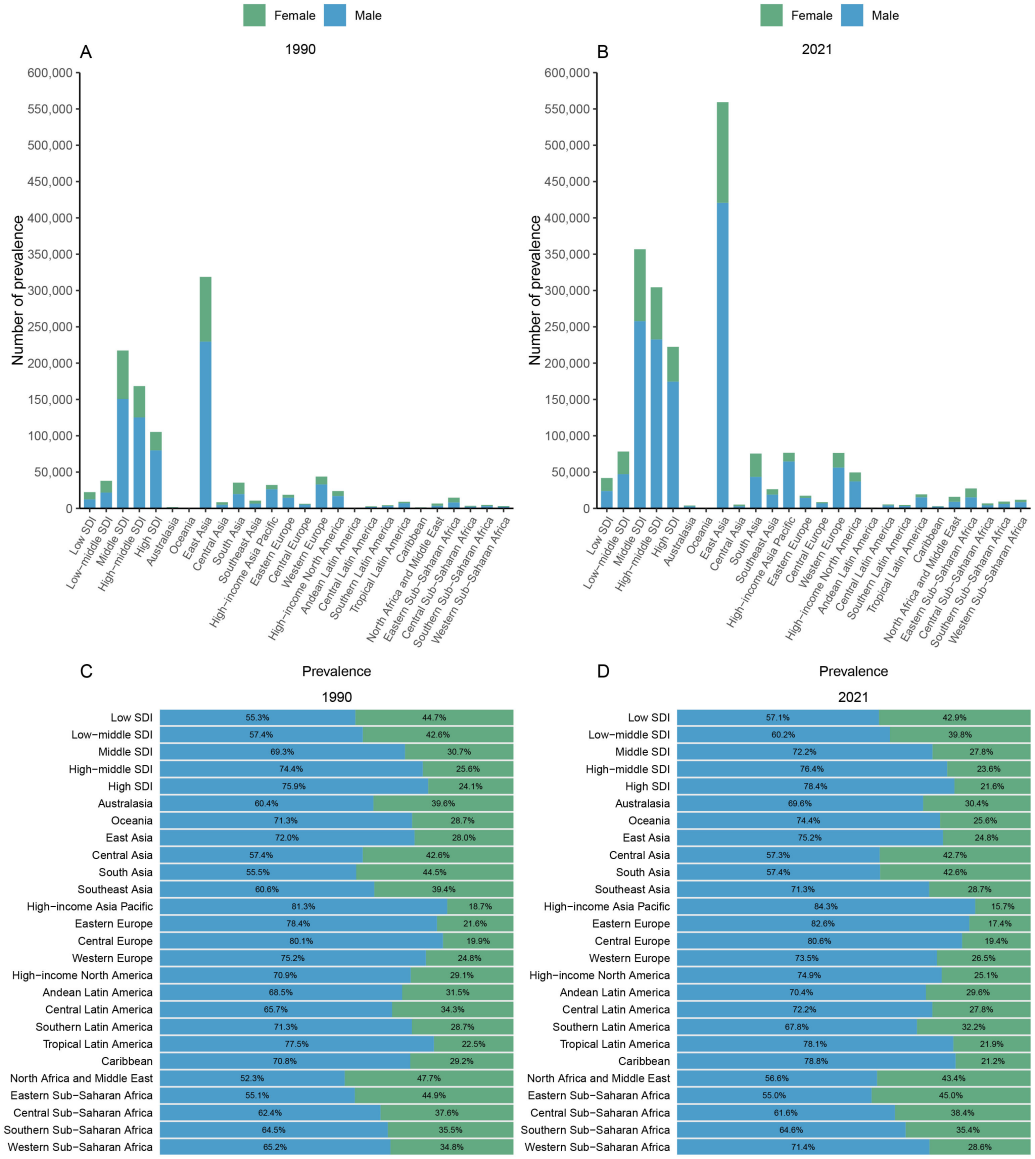
Gender disparity in esophageal cancer prevalence by GBD region: numbers in 1990 **(A)** and 2021 **(B)**, and proportional distributions in 1990 **(C)** and 2021 **(D)**.

Esophageal cancer cases, including prevalence, incidence, mortality, and DALYs, have risen in most GBD regions comparing data from 2021 and 1990. East Asia exhibits the most significant increases in these metrics (**Figure 3**; **Supplementary Figures S1**-**S3**). In contrast, Australasia, Andean Latin America, Oceania, and Caribbean regions report low levels of esophageal cancer across all indicators.

Males consistently show higher rates of esophageal cancer than females across all GBD regions. By 2021, the proportion of affected males is expected to increase in most regions compared to 1990, with High-income Asia Pacific region recording the highest rates among males (84.3%, 83.3%, 82.7%, and 84.6% for prevalence, incidence, mortality, and DALYs, respectively) (**Figure 3**; **Supplementary Figures S1**-**S3**).

**Figure 4:**
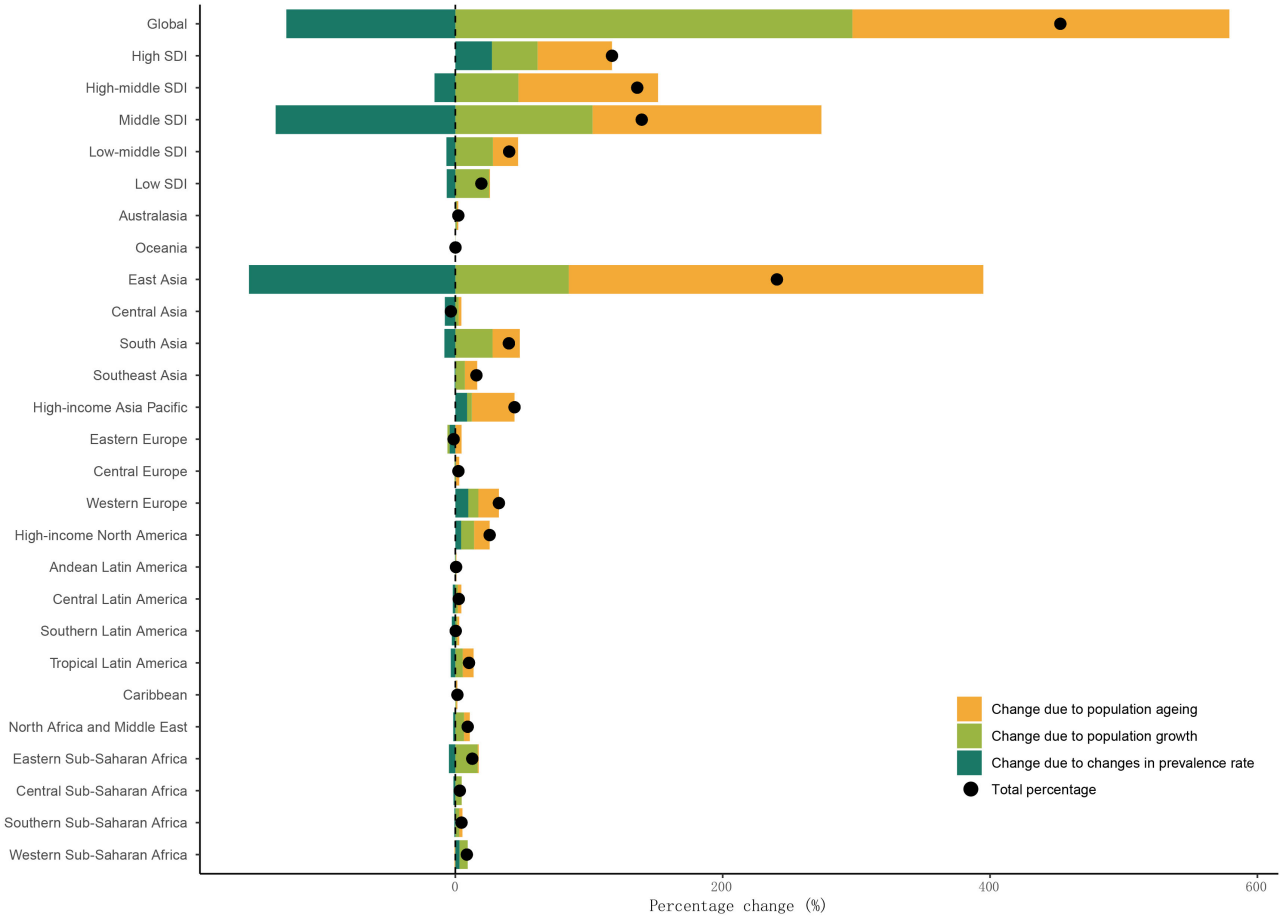
Population-level determinants of esophageal cancer: shifts in growth, ageing, and prevalence rates.

Decomposition analysis reveals the relative impacts of population growth, ageing, and shifts in prevalence, incidence, death, and DALY rates on the projected rise in cases globally and by GBD region (**Figure 4**; **Supplementary Figures S4**-**S6**). The global prevalence of esophageal cancer is primarily attributed to the overall population increase and ageing. However, changes in prevalence rates are the negative contributors to the total percentage in esophageal cancer cases (**Figure 4**). In East Asia, population growth, ageing and changes in prevalence rates are the main drivers of the global trends of esophageal cancer. The patterns for incidence and DALY prevalence mirror those of overall prevalence (**Supplementary Figures S4**, **S6**). Yet, changes in death rates, along with population growth and ageing, contribute to the rise in esophageal cancer deaths, with death rates changes being the most influential factor (**Supplementary Figure S5**).

### 3.3 Global disease burden of esophageal cancer from 1990 to 2021 according to age group

**Figure 5:**
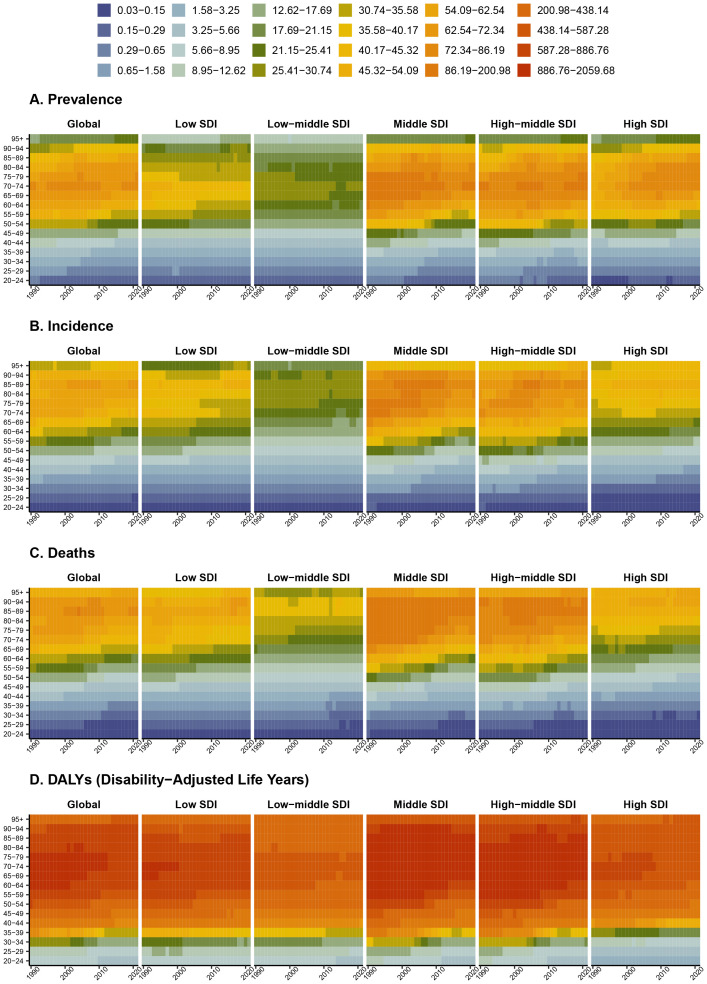
Variations in esophageal cancer burden across age groups and SDI regions. The Y-axis represents the burden (**(A)** prevalence, **(B)** incidence, **(C)** deaths, **(D)** Disability-Adjusted Life Years) across different age groups.

Between 1990 and 2021, a downward trend in esophageal cancer prevalence was observed across most age groups globally. Over these three decades, there has been a notable shift in the prevalence of esophageal cancer towards individuals over the age of 40. The rate is particularly high among those aged 60–79 in regions classified as middle, high-middle, and high SDI, whereas in low and low-middle SDI regions, the prevalence among the same age group is comparatively lower (**Figure 5A**). The incidence of esophageal cancer is relatively low in individuals under 40, with a significant increase of post-age 70. In middle SDI regions, the incidence rate for those over 60 exceeds that of other SDI regions (**Figure 5B**). Consistently, the mortality rate for esophageal cancer escalates sharply after the age of 40, reaching its zenith in the 85–89 age bracket. Here too, the middle SDI region exhibits a higher death rate compared to other regions (**Figure 5C**). The burden of esophageal cancer, measured in DALYs, escalates with age, accelerating particularly after age 40 and peaking between ages 60 to 90 (**Figure 5D**).

### 3.4 Global disease burden of esophageal cancer from 1990 to 2021 by country and territory

**Figure 6:**
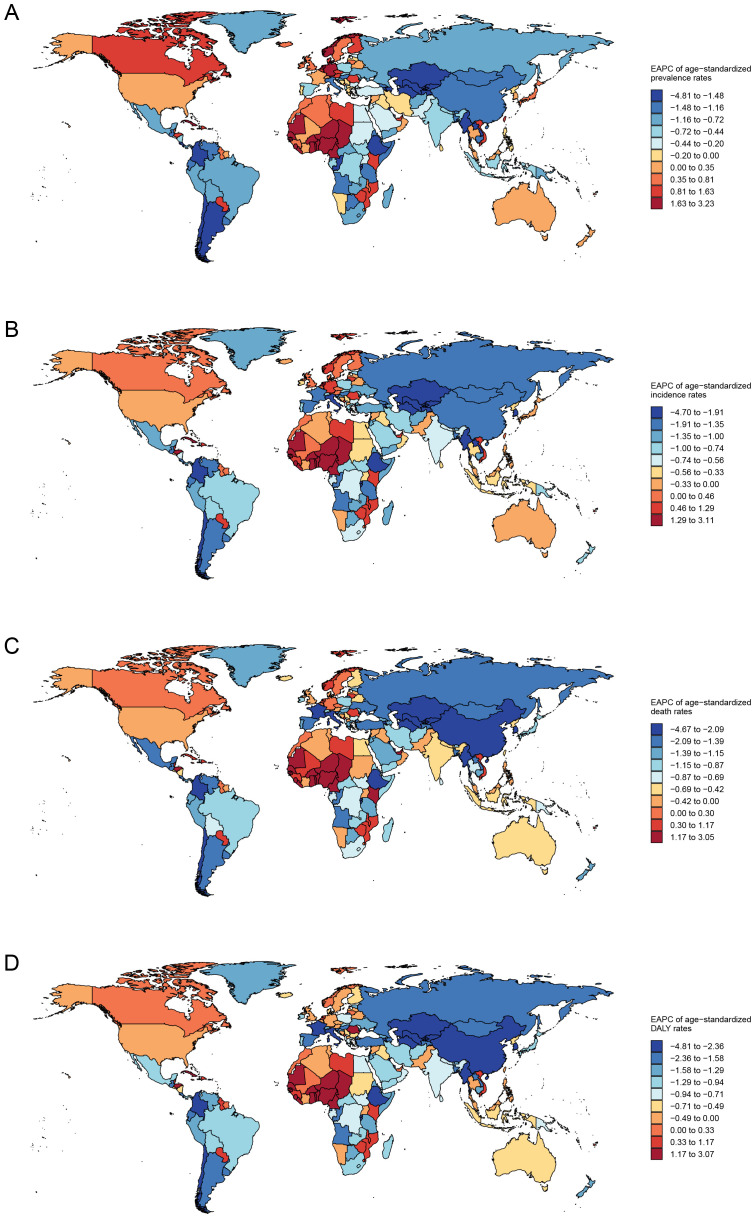
Global trends in esophageal cancer burden by country (**(A)** prevalence, **(B)** incidence, **(C)** deaths, **(D)** Disability-Adjusted Life Years).

Globally, the EAPC indicates a declining trend in the age-standardized prevalence rate of esophageal cancer across most countries. However, this downward trend is not uniform across all regions. Countries in West Africa, North Europe, and North America are exhibiting an increase in the prevalence rate of esophageal cancer (**Figure 6A**; **Supplementary Table S2**).

Similar upward trends are observed in age-standardized incidence, deaths, and DALY rates in West Africa and North America (**Figures 6B-D**). Notably, Uzbekistan, Kazakhstan, and Turkmenistan experience the most significant decline in mortality and DALY rates, with reductions of 4.7 (95% CI, 5.31 to 4.07), 4.55 (95% CI, 4.79 to 4.31), and 4.1 (95% CI, 4.64 to 3.56) respectively. The three countries exhibit the most significant downward trends in esophageal cancer mortality rates, with EAPC of -4.67 (95% CI, -5.3 to -4.05), -4.57 (95% CI, -4.83 to -4.31), and -4.12 (95% CI, -4.83 to -4.31). Correspondingly, the decreasing trends in DALY rates for these countries are 4.81 (95% CI, 5.4 to 4.21), 4.7 (95% CI, 4.92 to 4.47), and 4.13 (95% CI, 4.65 to 3.6) (**Figures 6B-D**; **Supplementary Tables S3**-**S5**).

### 3.5 Global analysis of the correlation between age-standardized rates and sociodemographic index in 2021

**Figure 7:**
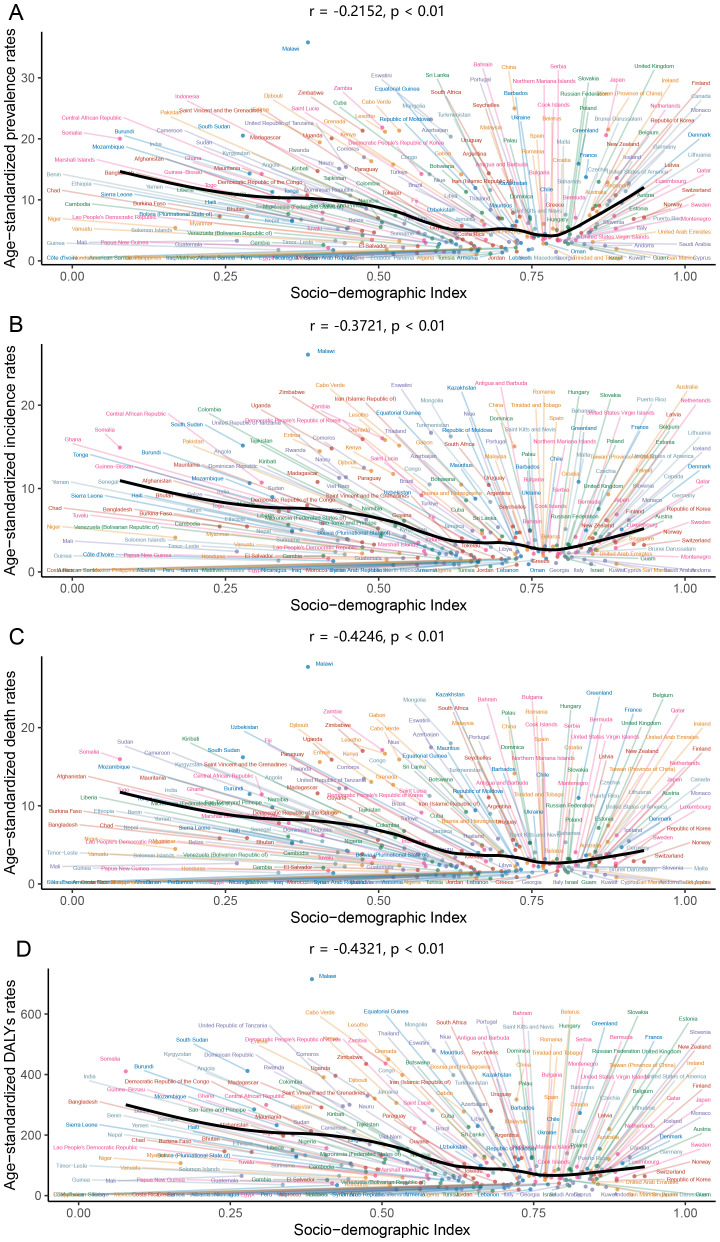
Correlation between disease burden and sociodemographic index for esophageal cancer (**(A)** prevalence, **(B)** incidence, **(C)** deaths, **(D)** Disability-Adjusted Life Years).

In the analysis of esophageal cancer’s global burden, a discernible pattern emerges as the SDI increases, the age-standardized rates of prevalence, incidence, mortality, and DALYs for esophageal cancer exhibit a decline. This suggests a positive correlation between high socio-economic status and improved health outcomes for esophageal cancer. However, the strength of these correlations varies, indicating that while there is a relationship, it is not starkly pronounced across all metrics. The correlation coefficients for prevalence, incidence, deaths, and DALYs are -0.2152, -0.3721, -0.4246, and -0.4321, respectively, each with a p-value less than 0.01 (**Figure 7**).

### 3.6 Health inequalities in esophageal cancer from 1990 to 2021

**Figure 8:**
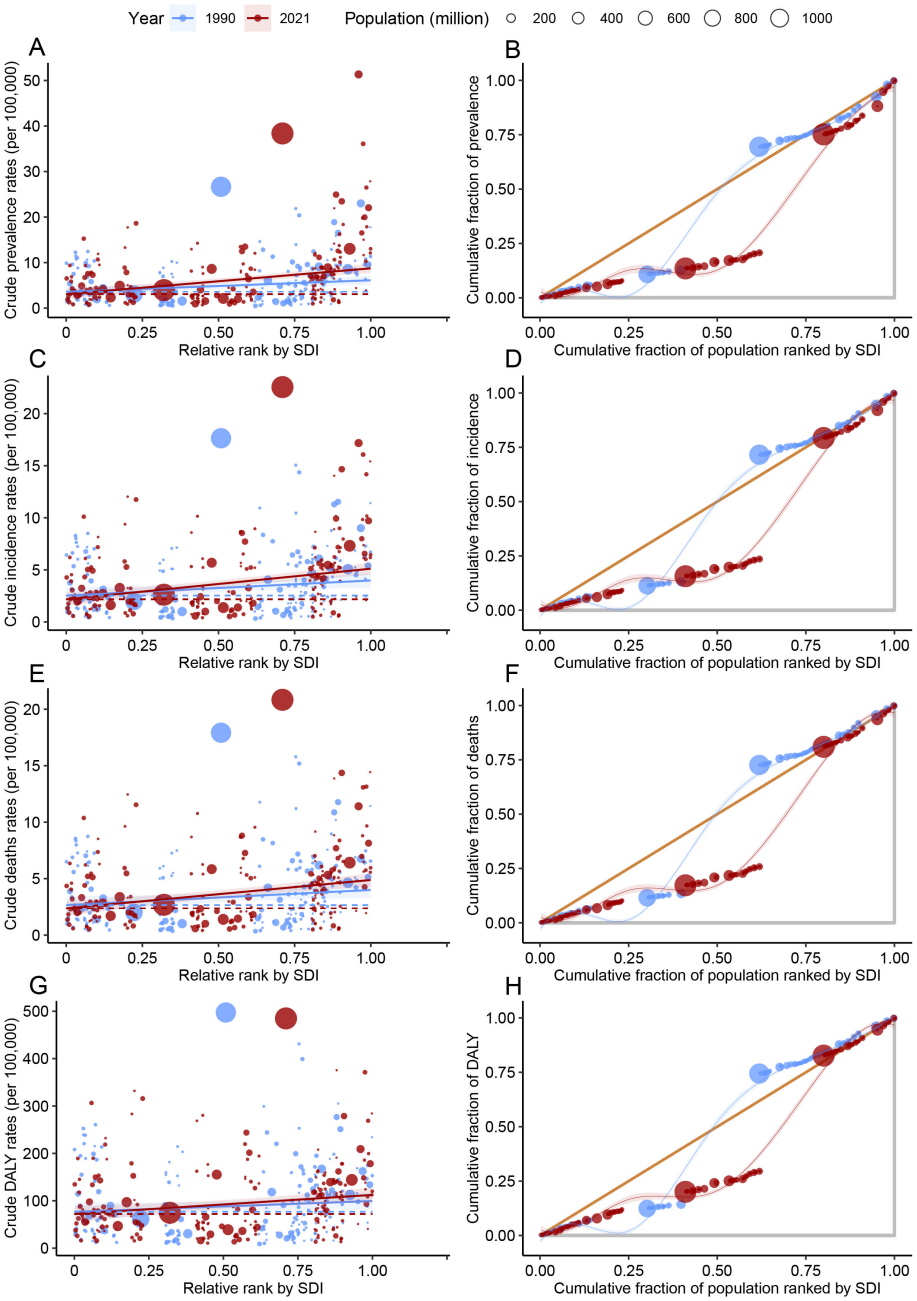
Assessing health inequality: regression and concentration curves for esophageal cancer disease burden. **(A, B)** prevalence; **(C, D)** incidence; **(E, F)** deaths; **(G, H)** DALYs. The x-axis and y-axis are defined in the figure.

Upon examining the esophageal cancer burden, it was determined that countries and territories with high SDI scores tend to carry a greater disease burden (**Figure 8**). The slope index of inequality (SII) indicates that the disparity in prevalence between the highest and lowest SDI countries and territories widened from 2.52 (95% CI, 1.19 to 5.01) in 1990 to 5.67 (95% CI, 5.34 to 10.67) in 2021. This trend is also observed in incidence, with the SII for deaths increasing from 1.45 (95% CI, 0.46 to 2.96) in 1990 to 2.94 (95% CI, 2.12 to 4.88) in 2021, and for DALYs, from 1.34 (95% CI, 0.30 to 2.86) in 1990 to 2.51 (95% CI, 1.52 to 4.10) in 2021. Similarly, the SII for absolute health inequality escalated from 22.72 (95% CI, -12.12 to 56.52) in 1990 to 40.31 (95% CI, 7.25 to 71.69) in 2021. Collectively, these findings signify an increase in absolute health inequality for esophageal cancer from 1990 to 2021. Furthermore, by 2021, esophageal cancer burden was more pronounced among higher social and economic strata.

## 4 Discussion

In this study, we conducted a comprehensive assessment of the global impact of esophageal cancer, stratified by sex, age, SDI, and geographical distribution. Prior research has begun to elucidate the burden of esophageal cancer, yet it has fallen short in pinpointing the specific contributors to this burden (6, 18). Most notably, there is a conspicuous absence of analysis on health disparities across countries with different SDI levels, which is crucial for a holistic understanding of esophageal cancer trends and the extensive disparities in global health.

Between 1990 and 2021, there was a notable increase in global metrics for esophageal cancer, including prevalence, incidence, mortality, and DALYs. Despite this, age-standardized rates showed a slight decline, likely due to the increase in total global population and the aging demographic (29). Projected trends indicate a continued decrease in these standardized rates through to 2050, with East Asia anticipated to experience the most significant reduction in patient numbers.

Projected trends indicate a decline in esophageal cancer disease burden, which may be partially attributed to the development of novel therapeutic strategies. Currently, the primary therapeutic modalities for esophageal cancer encompass surgery, radiation therapy, and chemotherapy (30). Over the past decade, the Food and Drug Administration (FDA) approving three types of targeted agents for esophageal cancer treatment. These include inhibitors targeting the epidermal growth factor receptor (EGFR) and vascular endothelial growth factor (VEGF), as well as monoclonal antibodies directed against the human epidermal growth factor receptor 2 (HER-2). Additionally, the PD-L1 inhibitor pembrolizumab has been sanctioned as a highly effective therapeutic option for patients with PD-L1-positive or advanced esophageal squamous cell carcinoma (ESCC) (31, 32). Despite these developments, several promising drugs remain under regulatory review. Nevertheless, over the past three decades, the clinical impact of these novel therapeutic approaches has been limited, with only modest improvements observed in overall survival rates compared to conventional treatments. Moving forward, the development of more effective therapeutic interventions and screen program remain imperative to address the unmet clinical needs in esophageal cancer management (33).

Worldwide, males consistently have higher rates of esophageal cancer in terms of prevalence, incidence, mortality, and DALYs compared to females. While prevalence declined across most age groups, there was a clear shift towards an older demographic, particularly among those aged 60–79 in mid to high SDI regions. Individuals under 40 showed low incidence and mortality rates, which increased sharply after age 70, with middle SDI regions showing higher rates. Although age-standardized prevalence rates generally decreased, high SDI countries and territories are facing an increasing disease burden, suggesting that the esophageal cancer burden decreases slowly with the increase in SDI.

Upon comparing various epidemiological studies on, distinct patterns have emerged: while the age-standardized incidence rates are on a global decline, regions with high SDI, such as North America and Europe, are experiencing an increasing disease burden. Gender disparities are also evident, with males consistently showing higher prevalence, incidence, mortality, and DALYs than females, and this disparity is expanding over time. Despite a decrease in esophageal cancer prevalence, the total number of cases is rising due to overall population growth and aging (34, 35). Furthermore, health inequalities persist even in higher socio-economic areas, indicating the complexity and diversity of health issues. These observations highlight the necessity for tailored public health strategies to tackle these challenges (36), especially in high SDI regions, and to consider shifts in gender and age demographics.

The higher incidence of esophageal cancer in male compared to female is primarily attributed to a combination of biological differences, lifestyle choices, and environmental influences (37, 38). Males are also more prone to engage in risky behaviors such as smoking and excessive alcohol consumption, which significantly contribute to the development of esophageal cancer (17, 38). Notably, China accounts for nearly half of the world’s esophageal cancer cases, with genetic tendencies, dietary practices like consuming hot foods and beverages, and socioeconomic factors being implicated (13–15).

In theory, health outcomes should improve with an increase in the Socio-Demographic Index (SDI). However, the rising burden of esophageal cancer may be linked to several factors. One contributing reason is population aging, as high-SDI regions tend to have older populations, and advancing age is associated with higher disease incidence (35). Another factor is the influence of risk factors. In Western and European developed countries, esophageal adenocarcinoma (AC) is the predominant type, while in developing regions such in “Asian Esophageal Cancer Belt” regions, squamous cell carcinoma (SCC) is more prevalent (39). Obesity, a major and consistent risk factor for AC, has become a significant public health concern in developed countries, and its increasing prevalence has driven the rise in esophageal cancer incidence in high-SDI regions (40, 41). On one hand, high SDI regions typically possess more advanced medical resources and diagnostic infrastructure, which facilitates the detection of more cancer cases. For instance, Japan has implemented a long-standing national endoscopic screening program for gastric cancer, aimed at early detection (42). However, in low-income settings the high cost and potential complications associated with endoscopic screening hinder its widespread implementation at the national level (43). These regions often face challenges such as limited medical resources, constrained budgets, and inadequate healthcare infrastructure, making large-scale screening programs difficult to establish. Additionally, lower health awareness and limited healthcare access among populations in low-income areas further reduce early cancer detection rates. Therefore, unless accompanied by the promotion of healthy lifestyles and effective control of risk factors, the burden of esophageal cancer is likely to continue rising despite advancements in socio-economic development.

Further investigation is essential to elucidate the epidemiological variations of esophageal cancer among different regions and demographic groups, with particular attention to the distinct characteristics of squamous cell carcinoma and adenocarcinoma, the two predominant histological subtypes. There is a necessity for additional research to assess how socioeconomic advancements influence the incidence of esophageal cancer and to determine the effectiveness of preventative strategies aimed at specific risk factors.

To effectively tackle the increasing burden and disparities in esophageal cancer, targeted policies should be implemented across both high-SDI and low-SDI regions. These policies should focus on precision prevention, early diagnosis, improved treatment, and the integration of equity metrics into national cancer control plans. High-SDI Regions:1) precision prevention targeting high-risk subgroups (obesity, aging populations, and males with a history of smoking). 2) Obesity: promote legislation to reduce processed meat consumption (e.g., warning labels) and subsidize fresh produce in food deserts. And develop and implement comprehensive fitness, weight loss, and health promotion programs. 3) GERD management: develop national guidelines for proton pump inhibitor (PPI) use and lifestyle modifications tailored to high-obesity populations. Low-SDI Regions:1) establish affordable and accessible screening programs, particularly in high-risk areas such as the “Asian Esophageal Cancer Belt.” 2) Strengthen health education initiatives to raise awareness about esophageal cancer risk factors and the importance of early detection. Global efforts: 1) increase investment in research and development of novel drugs and treatment methods to improve the efficacy and prognosis of esophageal cancer. 2) Incorporate equity metrics such as the SII and CI into national cancer control strategies. Ensure that policies are informed by decomposition and inequality analyses to address the root causes of disease burden and disparities.

The GBD 2021 study ensures data quality and addresses regional variability through a comprehensive and rigorous approach (https://www.healthdata.org/research-analysis/gbd). It integrates data from over 328,938 primary sources, covering 204 countries and territories, and provides highly standardized estimates of health outcomes and systems. The GBD 2021 study employs advanced statistical models and uncertainty analysis to account for variability and improve the accuracy of its estimates. Additionally, it leverages a global network of over 12,000 researchers from more than 160 countries to validate data sources and incorporate local expertise. This collaborative effort helps address regional differences and ensures that the data reflects diverse health trends and challenges worldwide. However, while these measures significantly improve data quality, ongoing efforts are necessary to address potential discrepancies and further refine the data to better reflect real-world health trends.

There were some limitations in our study.1) Data quality and reporting bias: the reliance on GBD 2021 data introduces potential biases, particularly in low- and middle-income regions where underreporting and misclassification of cases may occur due to limited diagnostic infrastructure and inconsistent cancer registry practices. 2) BAPC projections rely on historical data and do not account for future disruptions (e.g., pandemics, advancements in targeted therapies) that could alter disease trajectories. 3) Heterogeneity within subgroups: while disparities were analyzed by SDI, sex, and age, intra-regional variations (e.g., differences in healthcare access within high-SDI countries) were not fully explored. The study aggregated esophageal squamous cell carcinoma (ESCC) and adenocarcinoma (AC), two etiologically distinct subtypes, potentially masking subtype-specific risk factors and trends, due to insufficient data, a categorical analysis was not conducted. 4) Key determinants such as dietary habits, occupational exposures, and genetic predisposition were discussed but not quantitatively integrated into models due to data unavailability, limiting causal inference. 5) Health inequality metrics (SII, CI) focused on socioeconomic status but omitted cultural or behavioral factors that may independently influence outcomes. For the SII, we acknowledge its reliance on linear regression assumptions and sensitivity to model specification, while the CI’s interpretation may be influenced by extreme values or nonlinear distributions. We also highlight that the RCI, though useful for relative comparisons, may obscure absolute disparities. 6) Projection Uncertainties: long-term forecasts (to 2050) assume continuity in current prevention and treatment paradigms, which may not hold true if screening technologies or immunotherapy adoption accelerate unexpectedly.

## 5 Conclusion

Although projections indicate a declining trend, health disparities have intensified, with regions such as West Africa, Northern Europe, and North America experiencing an increasing prevalence of esophageal cancer. To mitigate these inequities, targeted interventions, improved healthcare accessibility, and preventive strategies in high-burden areas are imperative to alleviate the global burden and promote health equity. In high-SDI regions (e.g., North America, Western Europe), it is crucial to implement obesity control measures and integrate esophageal cancer screening into existing healthcare programs. In low-SDI regions (e.g., West Africa, South Asia), scaling up early detection initiatives and community education programs to reduce exposure to carcinogens is essential. Additionally, tobacco and alcohol control policies should be prioritized, particularly targeting males. Examples of such strategies include gender-specific cessation campaigns and stricter regulation of occupational carcinogen exposure in male-dominated industries. This approach underscores the importance of region-specific, evidence-based interventions to address the multifaceted challenges of esophageal cancer and advance global health equity.

## Data Availability

GBD study 2021 data resources were available online from the Global Health Data Exchange (GHDx) query tool (http://ghdx.healthdata.org/gbd-results-tool).

## Supplementary material

Supplementary Figure 1 Gender disparity in esophageal cancer incidence by GBD region: numbers in 1990 **(A)** and 2021 **(B)**, and proportional distributions in 1990 **(C)** and 2021 **(D)**.

Supplementary Figure 2 Gender disparity in esophageal cancer deaths by GBD region: numbers in 1990 **(A)** and 2021 **(B)**, and proportional distributions in 1990 **(C)** and 2021 **(D)**.

Supplementary Figure 3 Gender disparity in esophageal cancer DALYs by GBD region: numbers in 1990 **(A)** and 2021 **(B)**, and proportional distributions in 1990 **(C)** and 2021 **(D)**.

Supplementary Figure 4 Population-level determinants of esophageal cancer: shifts in growth, ageing, and incidence rates.

Supplementary Figure 5 Population-level determinants of esophageal cancer: shifts in growth, ageing, and death rates.

Supplementary Figure 6 Population-level determinants of esophageal cancer: shifts in growth, ageing, and DALY rates.
