# Supplementary material for: Global trends in esophageal cancer: sex and age disparities in health inequalities from 1990 to 2021, with projections to 2050
Source: Front Oncol. 2025 Jun 24;15:1563570. doi: 10.3389/fonc.2025.1563570 (PMC12234333; doi:10.3389/fonc.2025.1563570)
Supplement: Supplementary file 2 [file Table6.docx]

| **ICD-9** |
| --- |
| **150.0**: Malignant neoplasm of the upper part of the esophagus |
| **150.1**: Malignant neoplasm of the middle part of the esophagus |
| **150.2**: Malignant neoplasm of the lower part of the esophagus |
| **150.3**: Malignant neoplasm of the esophagogastric junction |
| **150.8**: Malignant neoplasm of other specified parts of the esophagus |
| **150.9**: Malignant neoplasm of the esophagus, unspecified site |
| **ICD-10** |
| C15.3: Malignant neoplasm of the upper third of the esophagus. |
| C15.4: Malignant neoplasm of the middle third of the esophagus. |
| C15.5: Malignant neoplasm of the lower third of the esophagus. |
| C15.9: Malignant neoplasm of the esophagus, unspecified. |
| C15.3: Malignant neoplasm of the upper third of the esophagus. |
| D00.1**：**Carcinoma in situ of the esophagus |
