# Supplementary figures and images for: Global trends in esophageal cancer: sex and age disparities in health inequalities from 1990 to 2021, with projections to 2050
Source: Front Oncol. 2025 Jun 24;15:1563570. doi: 10.3389/fonc.2025.1563570 (PMC12234333; doi:10.3389/fonc.2025.1563570)

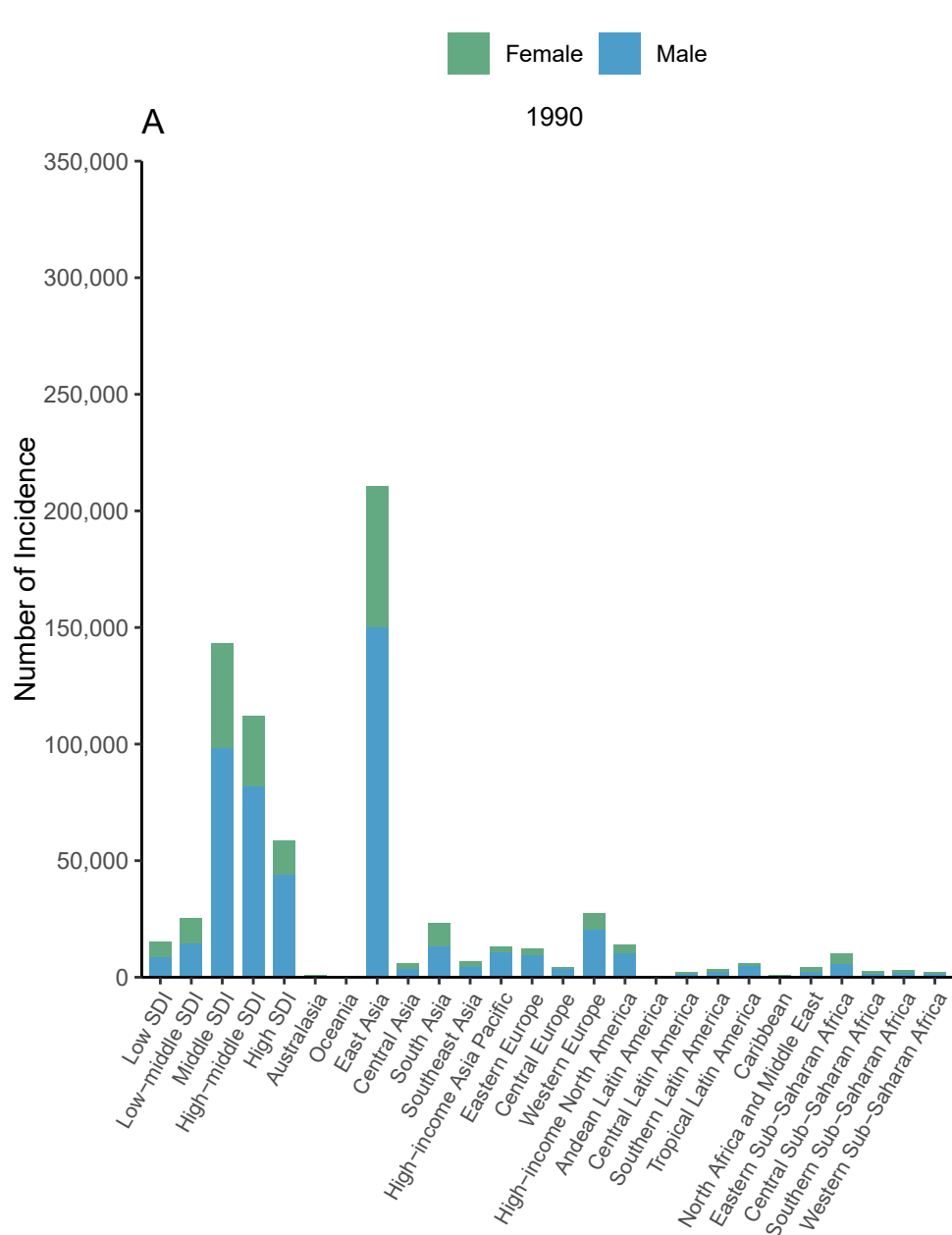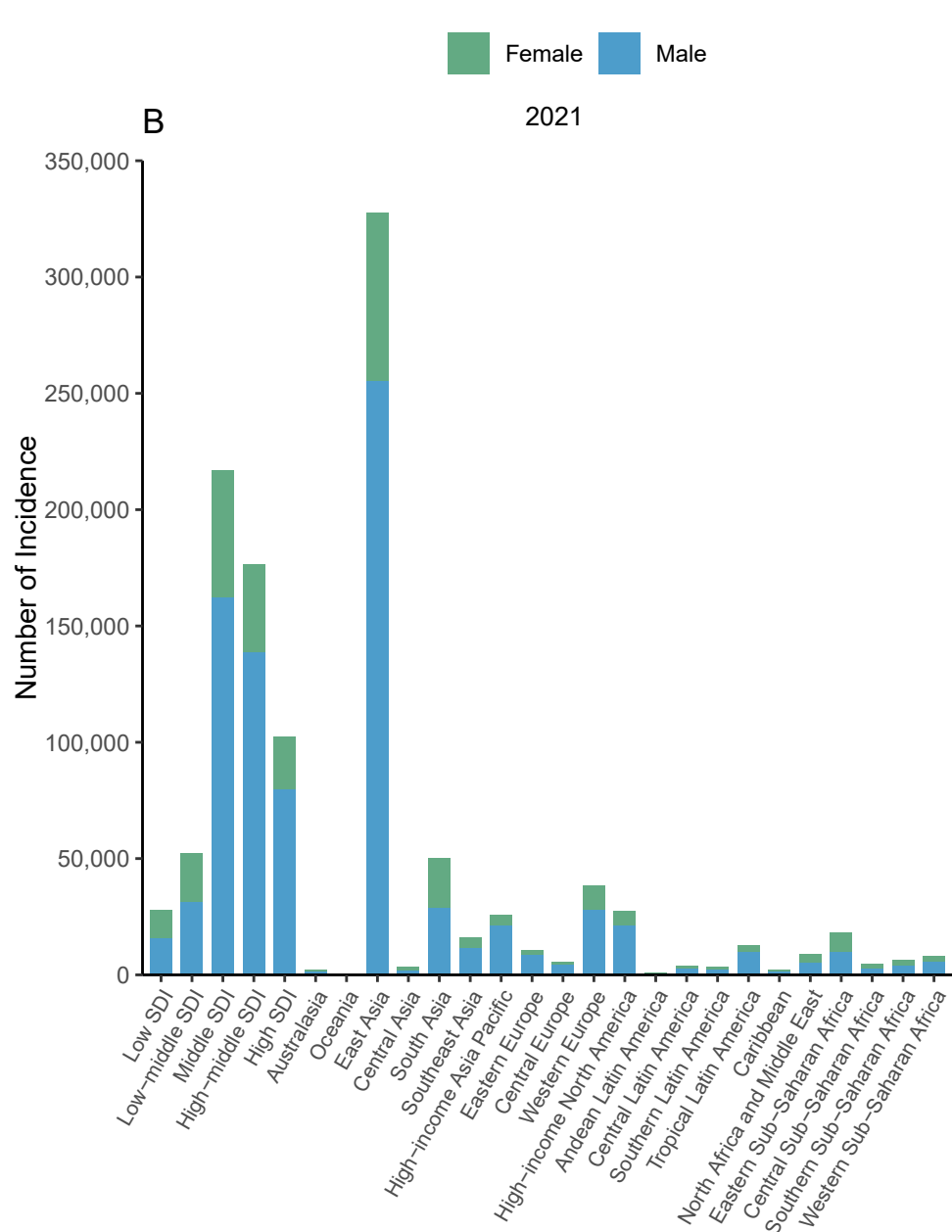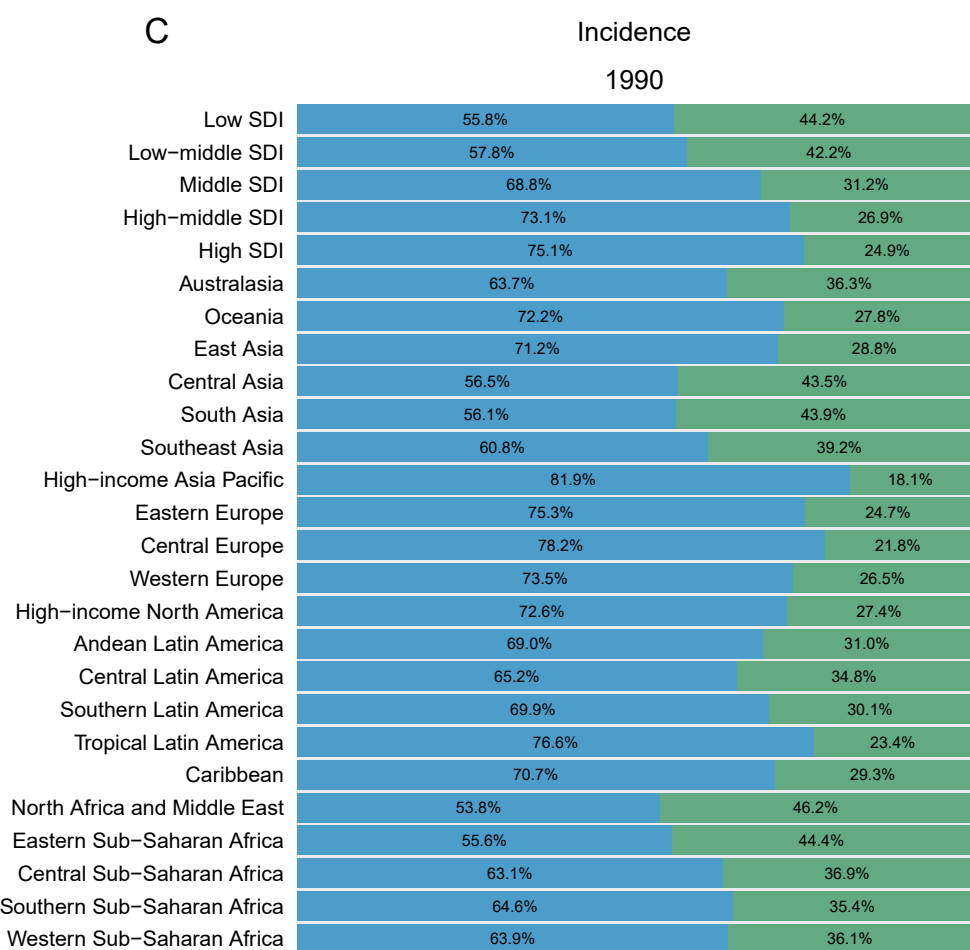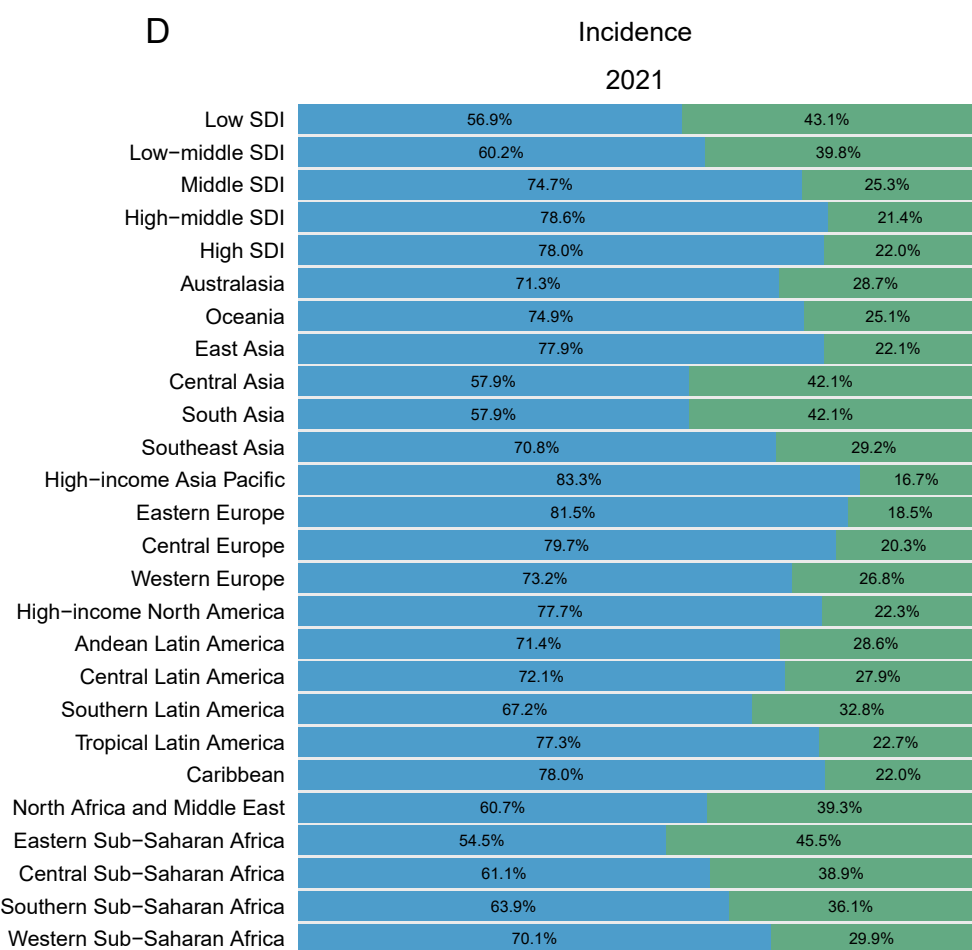

Supplement: Supplementary Figure 1 — Gender disparity in esophageal cancer incidence by GBD region: numbers in 1990 (A) and 2021 (B), and proportional distributions in 1990 (C) and 2021 (D). [file DataSheet1.pdf]

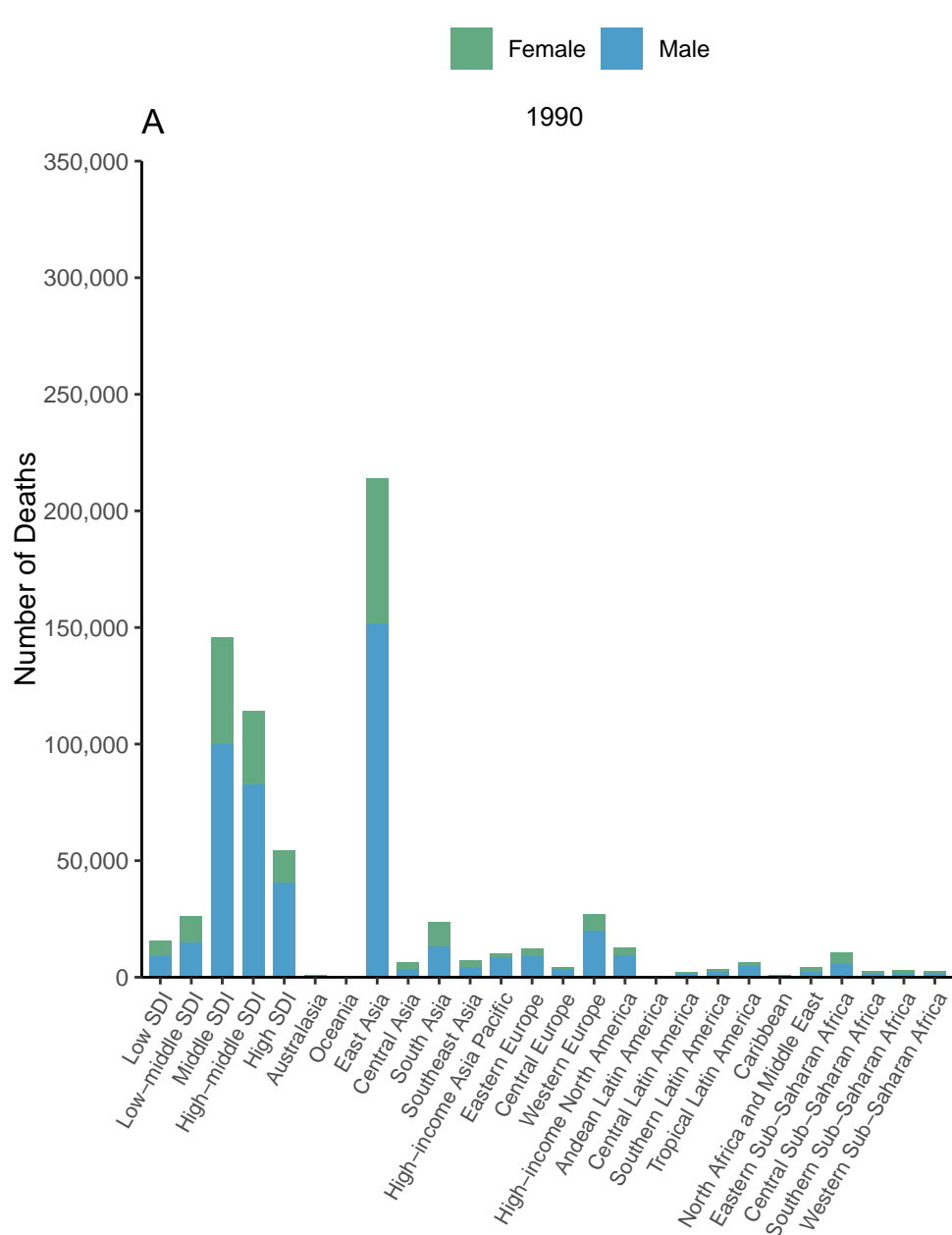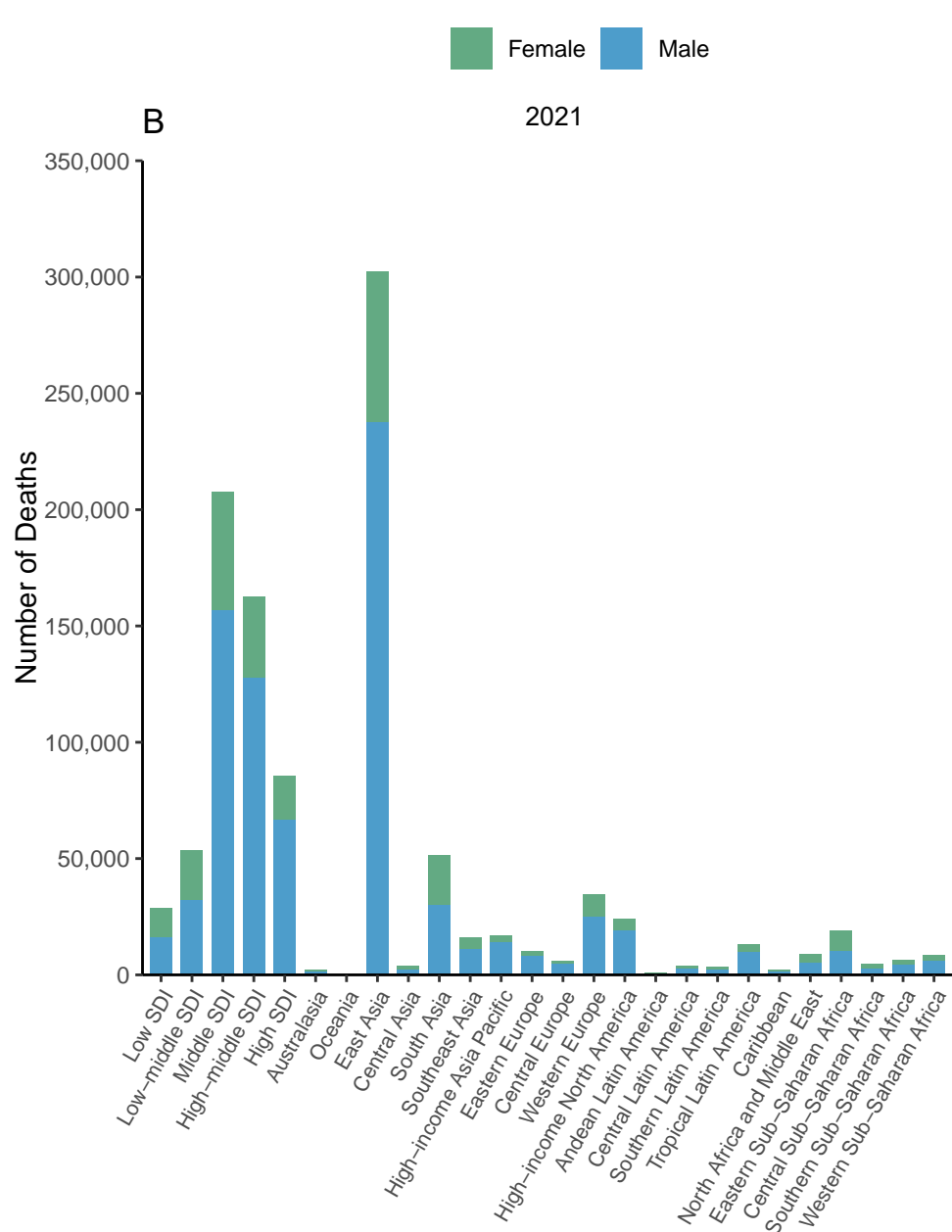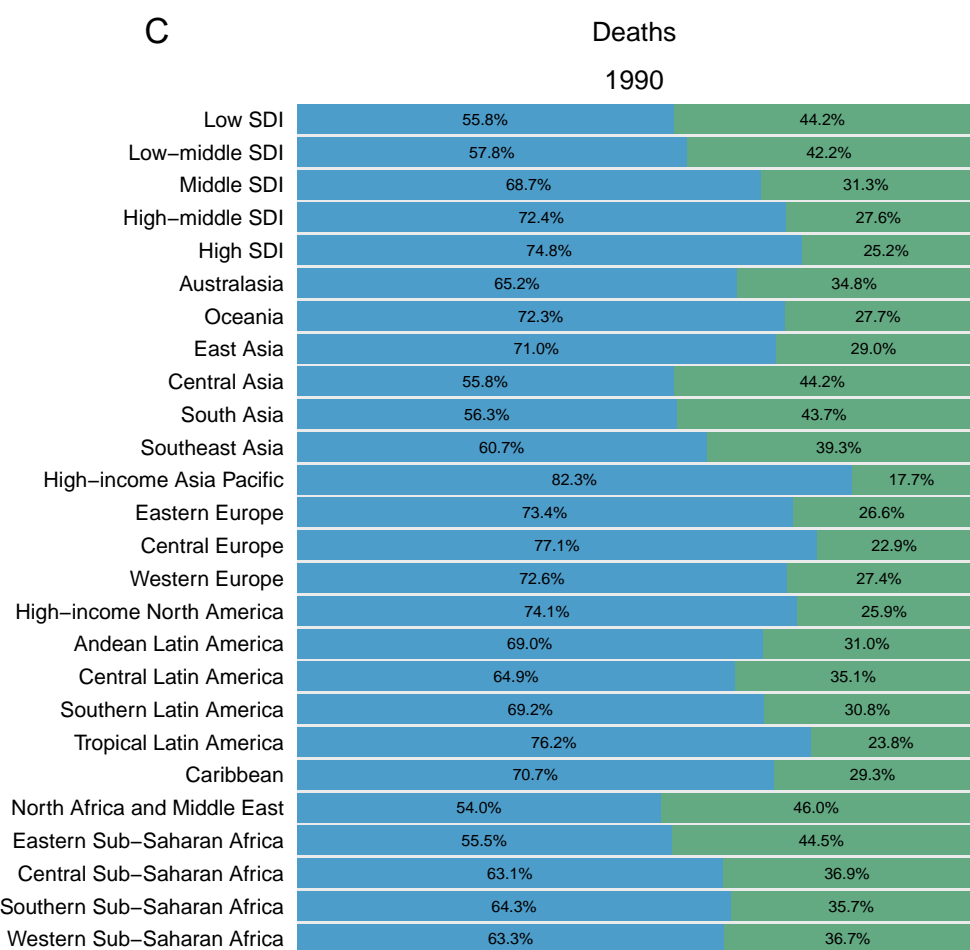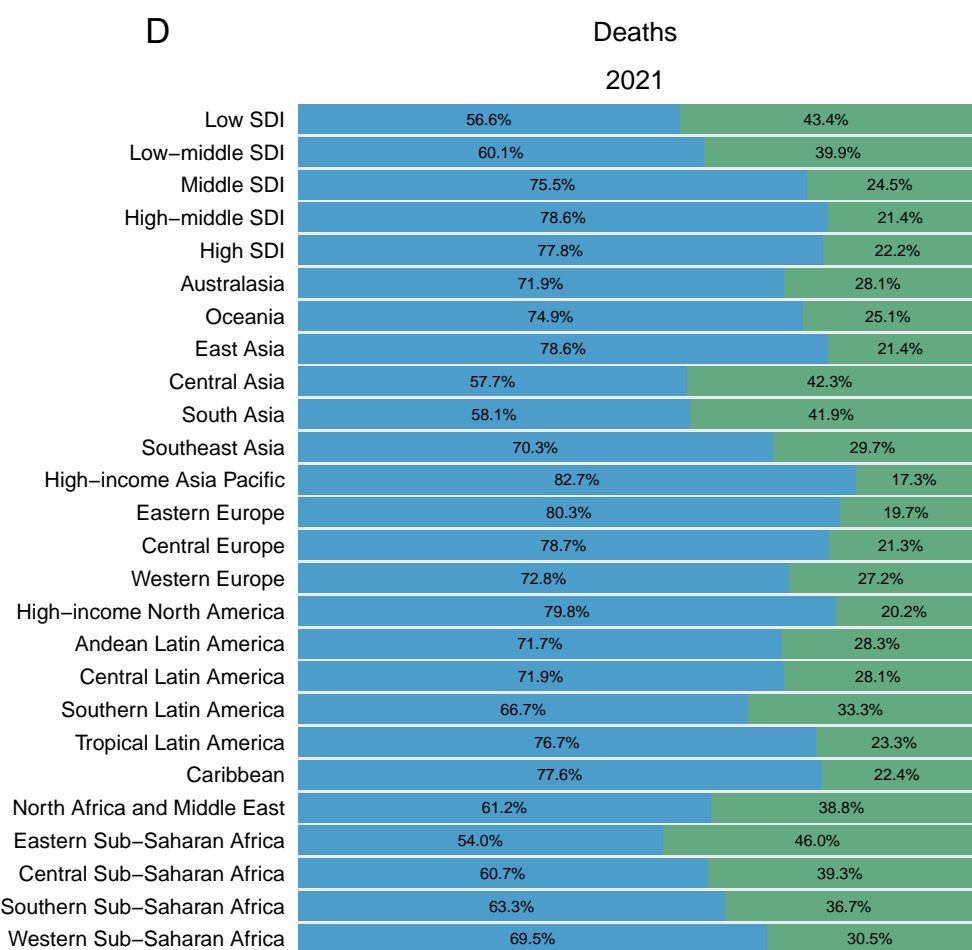

Supplement: Supplementary Figure 2 — Gender disparity in esophageal cancer deaths by GBD region: numbers in 1990 (A) and 2021 (B), and proportional distributions in 1990 (C) and 2021 (D). [file DataSheet2.pdf]

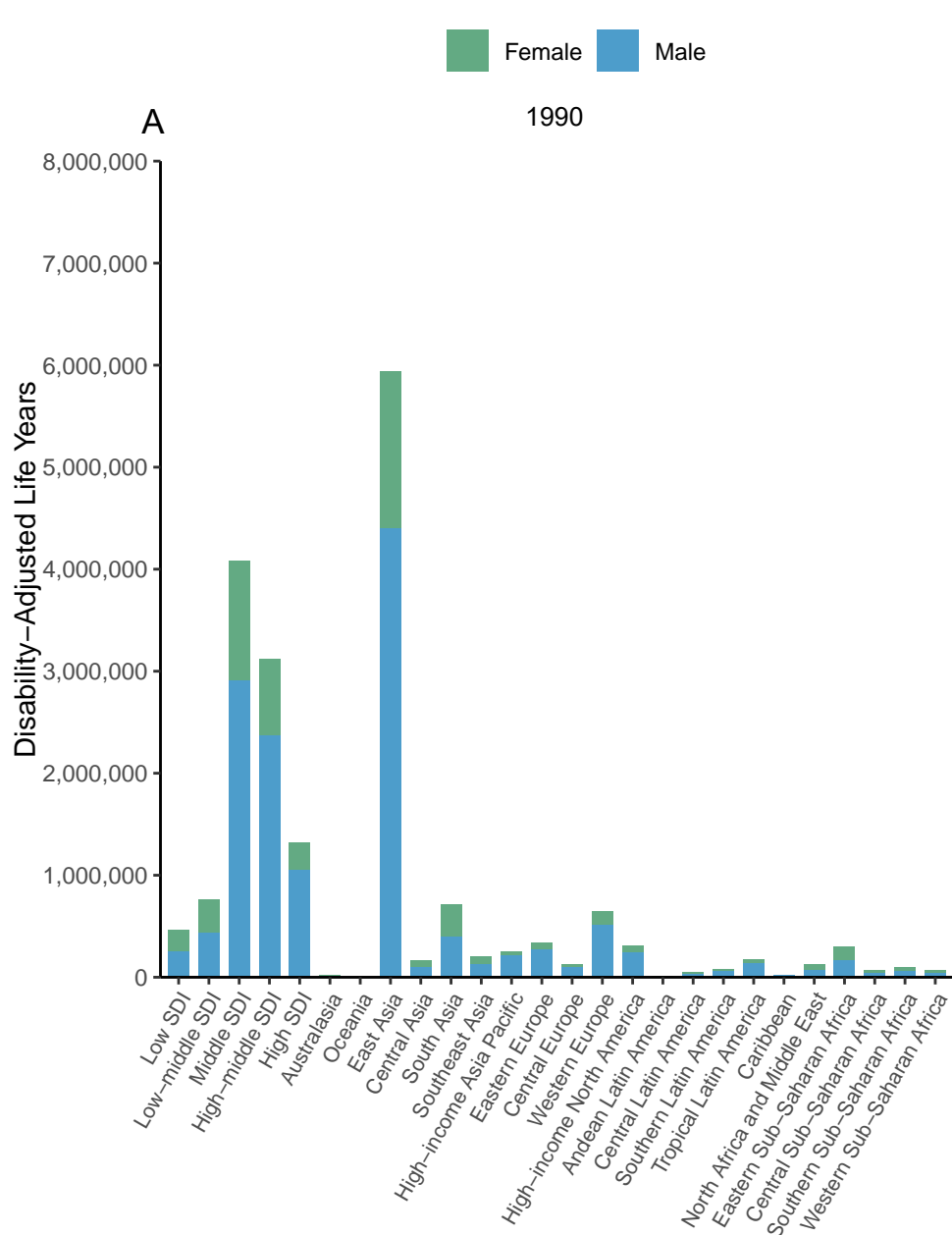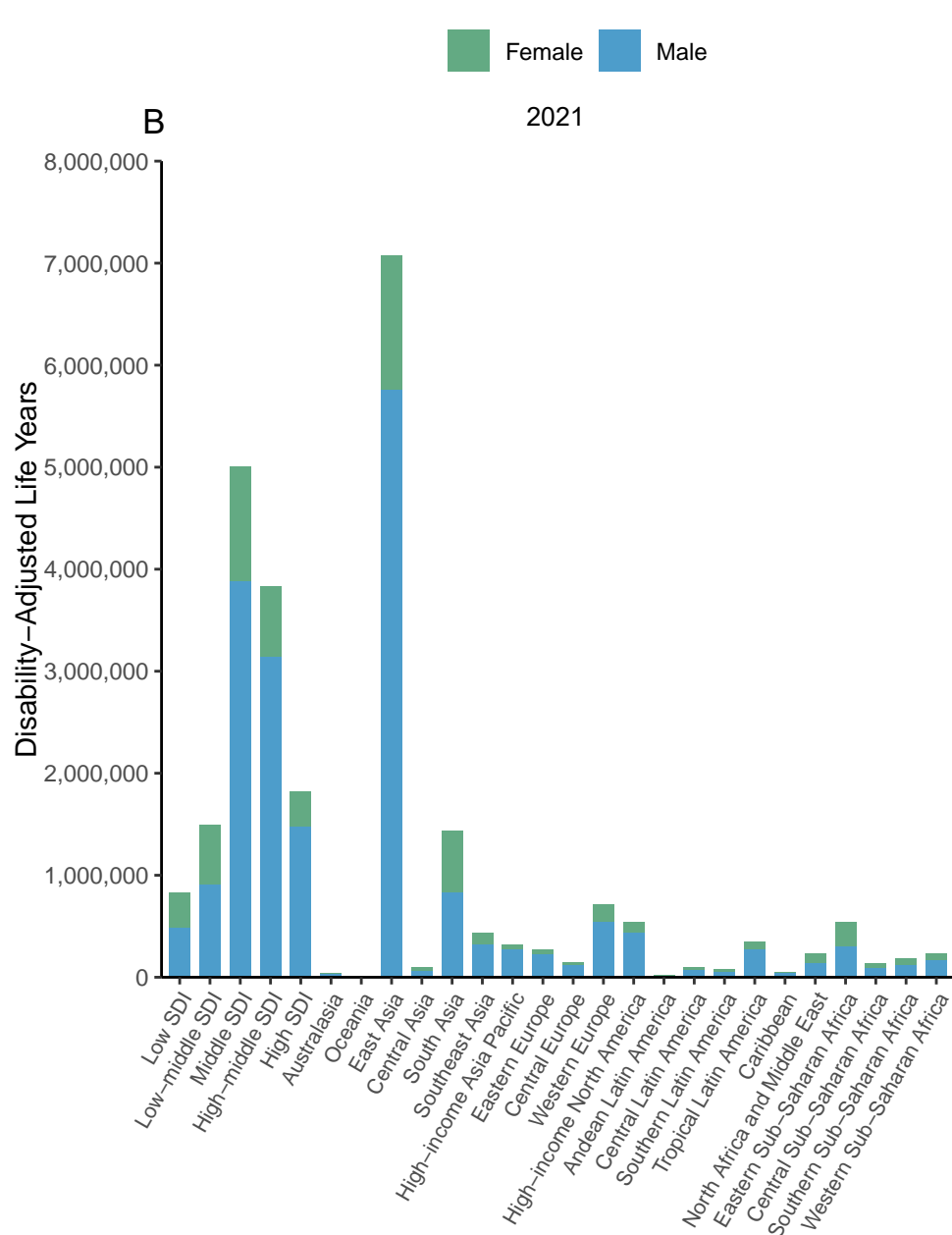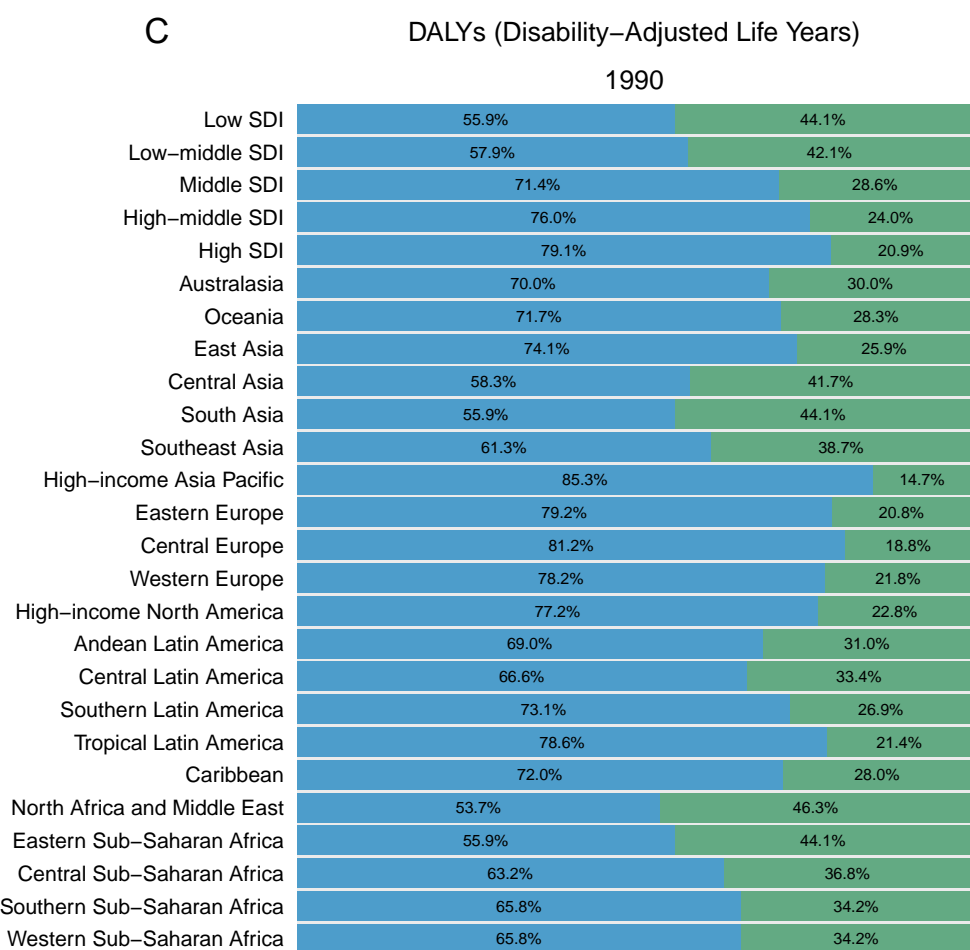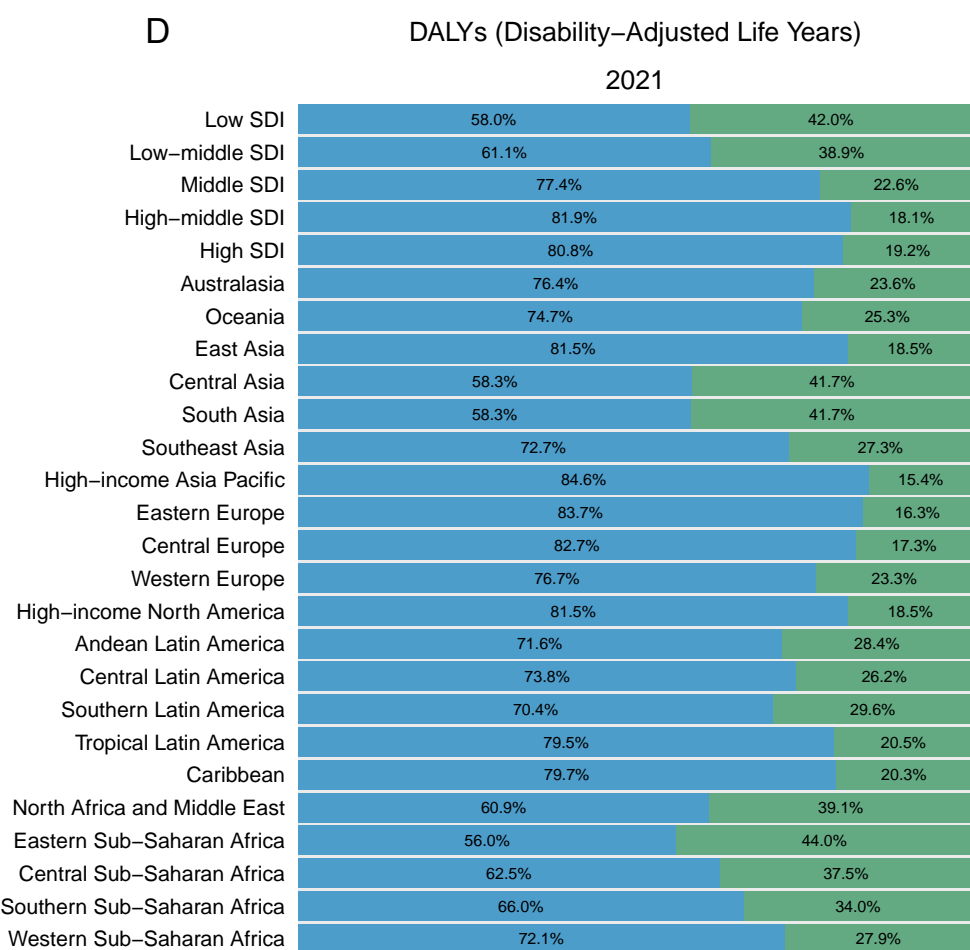

Supplement: Supplementary Figure 3 — Gender disparity in esophageal cancer DALYs by GBD region: numbers in 1990 (A) and 2021 (B), and proportional distributions in 1990 (C) and 2021 (D). [file DataSheet3.pdf]

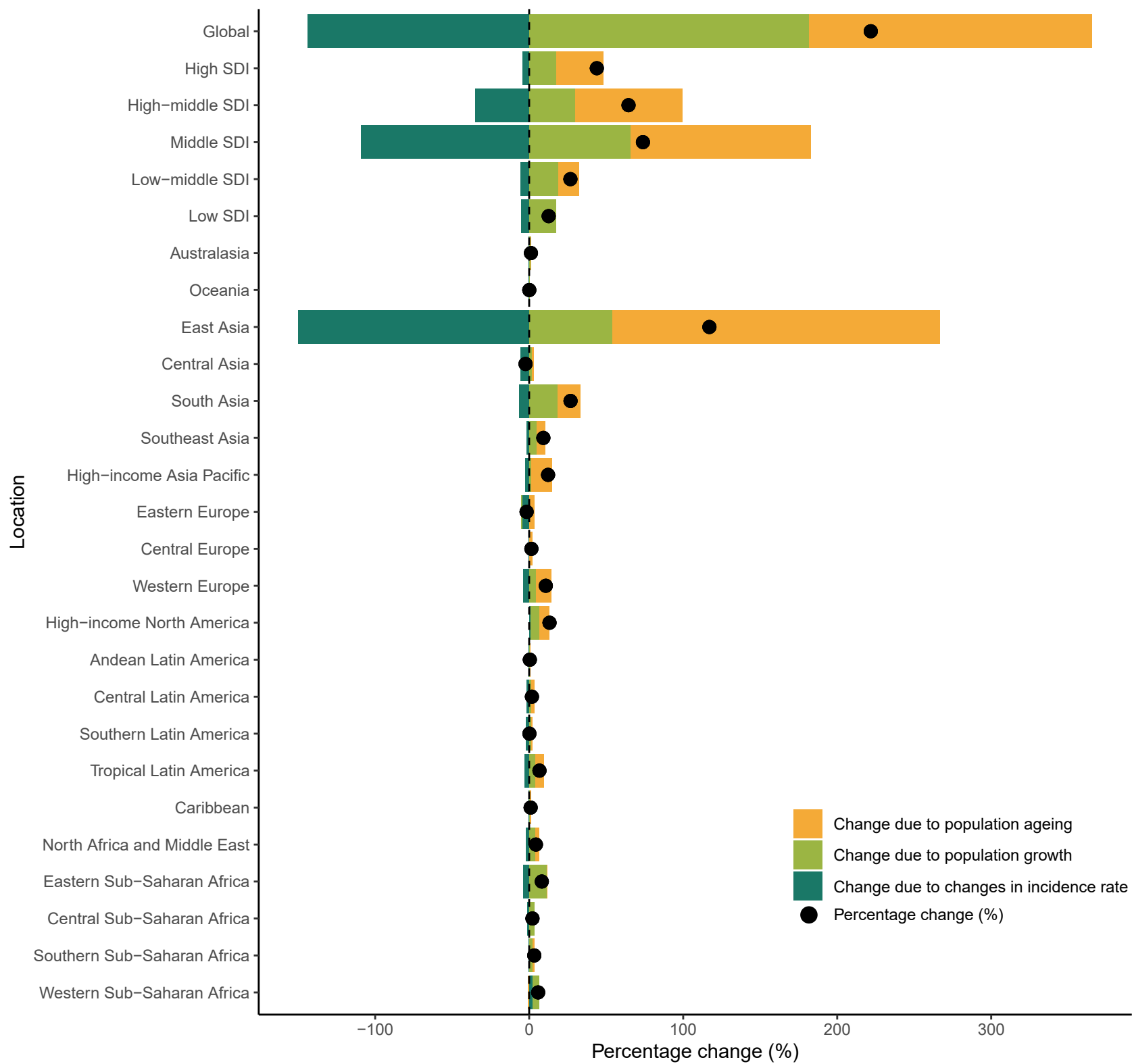

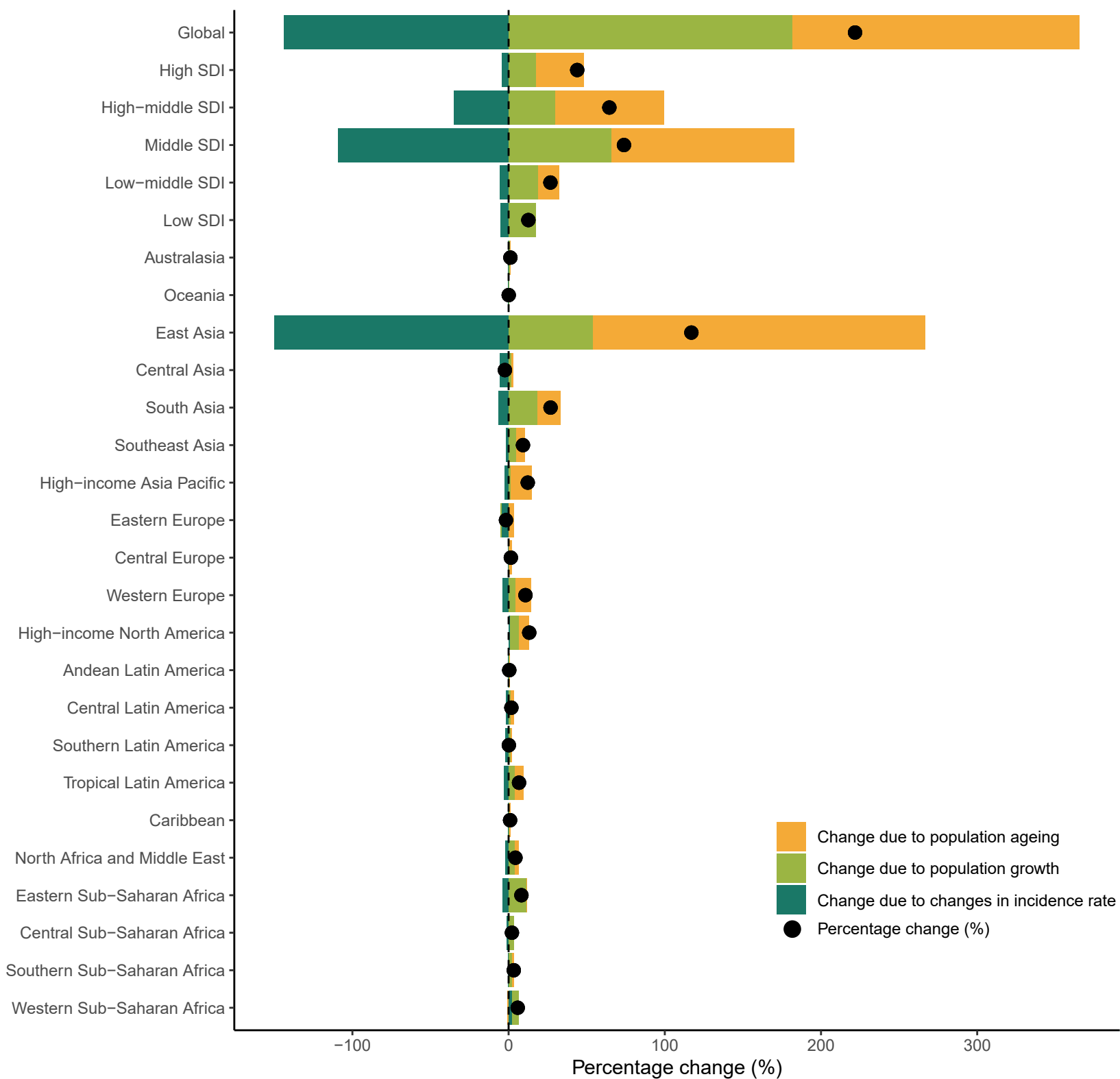

Supplement: Supplementary Figure 4 — Population-level determinants of esophageal cancer: shifts in growth, ageing, and incidence rates. [file DataSheet4.pdf]

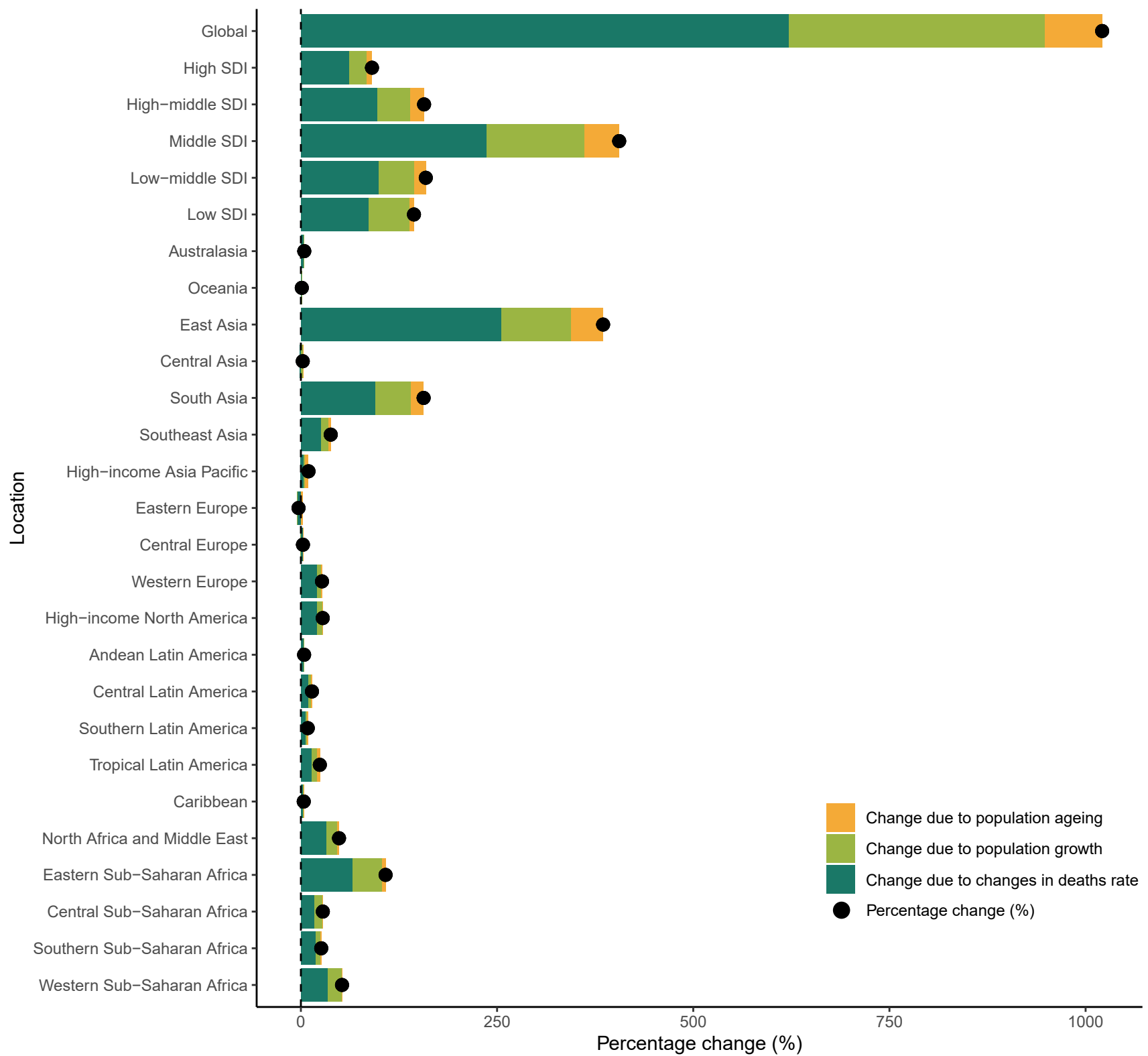

Supplement: Supplementary Figure 5 — Population-level determinants of esophageal cancer: shifts in growth, ageing, and death rates. [file DataSheet5.pdf]

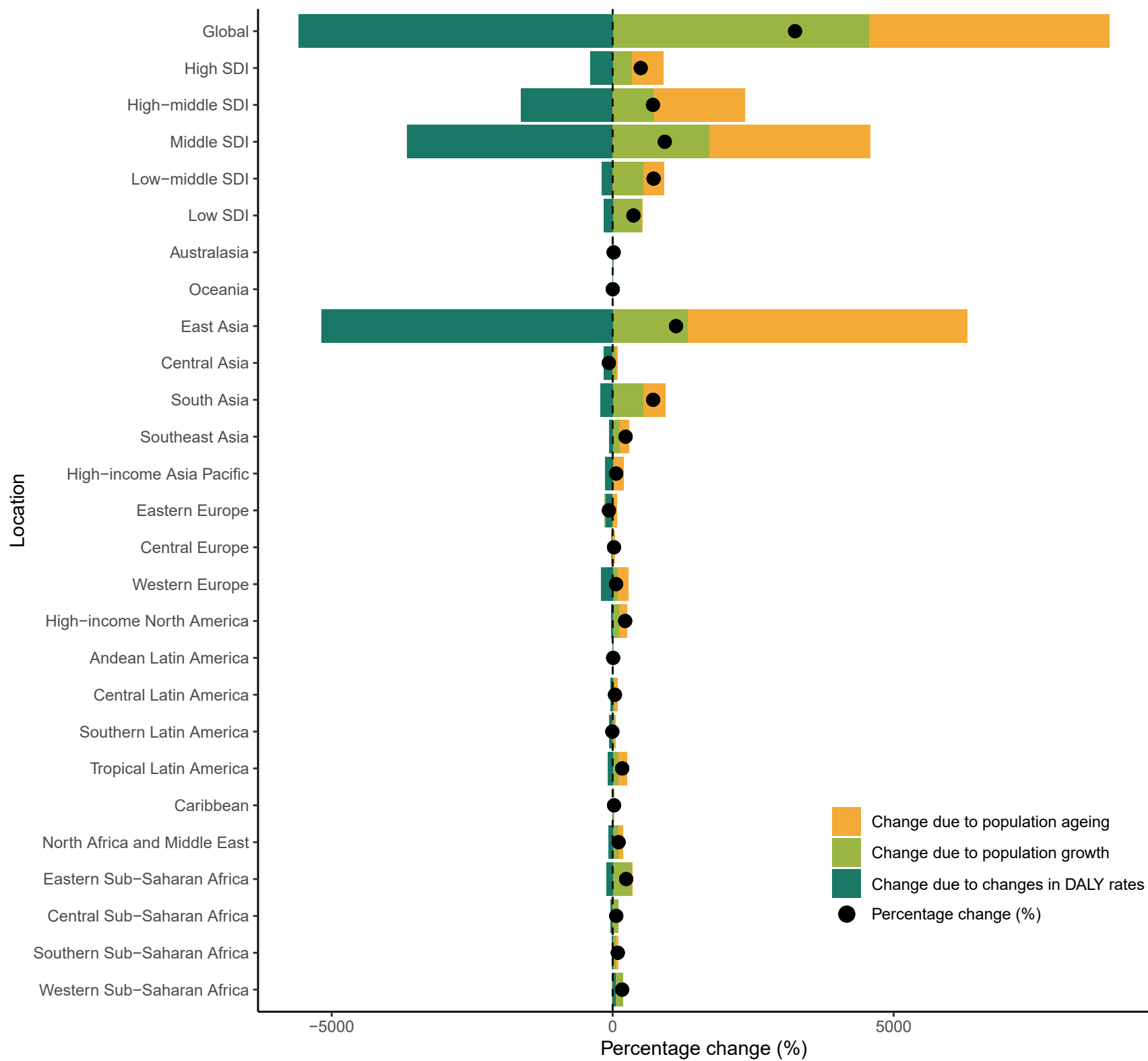

Supplement: Supplementary Figure 6 — Population-level determinants of esophageal cancer: shifts in growth, ageing, and DALY rates. [file DataSheet6.pdf]
